# Upregulation of long noncoding RNA HOXA-AS3 promotes tumor progression and predicts poor prognosis in glioma

**DOI:** 10.18632/oncotarget.18162

**Published:** 2017-05-24

**Authors:** Fan Wu, Chuanbao Zhang, Jinquan Cai, Fan Yang, Tingyu Liang, Xiaoyan Yan, Haoyuan Wang, Wen Wang, Jing Chen, Tao Jiang

**Affiliations:** ^1^ Department of Neurosurgery, Beijing Tiantan Hospital, Capital Medical University, Beiijing, China; ^2^ Department of Molecular Neuropathology, Beijing Neurosurgical Institute, Capital Medical University, Beiijing, China; ^3^ Department of Neurosurgery, The Second Affiliated Hospital of Harbin Medical University, Harbin, China; ^4^ Department of Neurosurgery, Zhujiang Hospital, Southern Medical University, Guangzhou, China; ^5^ Department of Neurosurgery, The Second Affiliated Hospital of Soochow University, Suzhou, China; ^6^ Center of Brain Tumor, Beijing Institute for Brain Disorders, Beiijing, China; ^7^ China National Clinical Research Center for Neurological Diseases, Beiijing, China; ^8^ Chinese Glioma Genome Atlas Network (CGGA), Beiijing, China

**Keywords:** glioma, LncRNA, HOXA-AS3, proliferation, tumorigenesis

## Abstract

Long noncoding RNAs (lncRNAs) have recently emerged as new potentially promising therapeutic targets in many cancers. However, their prognostic value and biological functions associated with glioma remain to be elucidated. Here, High-throughput RNAseq was performed to detect the expression profiles of lncRNAs in 325 human glioma tissues. It was shown that a novel lncRNA HOXA-AS3 was one of the most significantly upregulated lncRNAs in glioma tissues. Quantitative PCR further verified the increased expression of HOXA-AS3 in patient samples and glioma cell lines. Uni and Multivariate Cox regression analysis revealed that HOXA-AS3 was an independent prognostic factor in glioma patients. Gene set enrichment analysis indicated that the gene sets correlated with HOXA-AS3 expression were involved in cell cycle progression and E2F targets. Functionally, HOXA-AS3 silencing resulted in proliferation arrest by altering cell cycle progression and promoting cell apoptosis, and impaired cell migration in glioma cells. Furthermore, the growth-inhibiting effect of HOXA-AS3 knockdown was also demonstrated in Xenograft mouse model. Our results highlight the important role of HOXA-AS3 in glioma progression, and indicate that HOXA-AS3 may be served as a valuable prognostic biomarker for glioma.

## INTRODUCTION

Gliomas are the most common and malignant primary brain tumors, accounting for 70% of adult malignant primary brain tumors, and the yearly incidence is approximately 6 cases per 100000 [1]. Despite all surgical efforts in combination with chemo and radiotherapy, gliomas are still incurable, and the prognosis remains dismal. The median overall survival of Glioblastoma (GBM) which is the most aggressive glioma is less than 15 months, and the 5-year survival rate of patients with GBM is less than 3% [2]. The progress of treatments for glioma is hampered due to the infiltrative growth and inherent resistance to both chemo and radiotherapy [3]. To better understand and to find more effective treatments for this disease, it is vital to identify novel biomarkers and therapeutic targets.

Recently, it is becoming apparent that only a small percentage (1–2%) of the whole human genome encodes proteins, whereas the majority of the genome encodes large numbers of noncoding RNAs [4]. LncRNAs are a class of noncoding transcripts longer than 200 nucleotides without protein-coding potential [5]. Increasing evidence have demonstrated that lncRNAs play essential roles in large range of cellular processes, including proliferation, metastasis, differentiation and stem cell pluripotency though epigenetic modification and chromatin remodeling [6]. Furthermore, lots of studies have shown that several lncRNAs are deregulated in many tumors and involve in cancer development and progression [7], for example, lncRNA HOTAIR in breast and colorectal cancer [8, 9], MALAT1 in lung adenocarcinoma and liver cancer [10, 11], H19 in bladder cancer and lung cancer [12, 13], and MEG3 in leukemia [14]. In the case of glioma, lncRNA XIST exerts tumor-suppressive functions by up-regulating miR-152 in glioblastama stem cells [15]. CRNDE plays an oncogenic role of glioma stem cell through the negative regulation of miR-186 [16]. Our previous study revealed that HOTAIR is a cell cycle-associated lncRNA and servers as a prognostic factor for glioma patient survival [17]. Hence, to identify more cancer associated lncRNAs and to investigate their biological functions and underlying mechanisms are critical for better clarifying the molecular biology of glioma.

In this study, we identified a new LncRNA-HOXA-AS3, which is 3992 nt in length and located in chromosome 7p15.2. HOXA-AS3 was upregulated in glioma tissues and cells, and its high-expression was associated with tumor grade and poor prognosis in patients. Furthermore, *in vitro* and *in vivo* knockdown assays were performed to determine the functions of HOXA-AS3 in glioma tumorigenesis and progression. Thus, our study has identified a novel lncRNA, HOXA-AS3, which could be a potential therapeutic target for glioma.

## RESULTS

### HOXA-AS3 expression is upregulated in glioma tissues and cell lines

**Figure 1:**
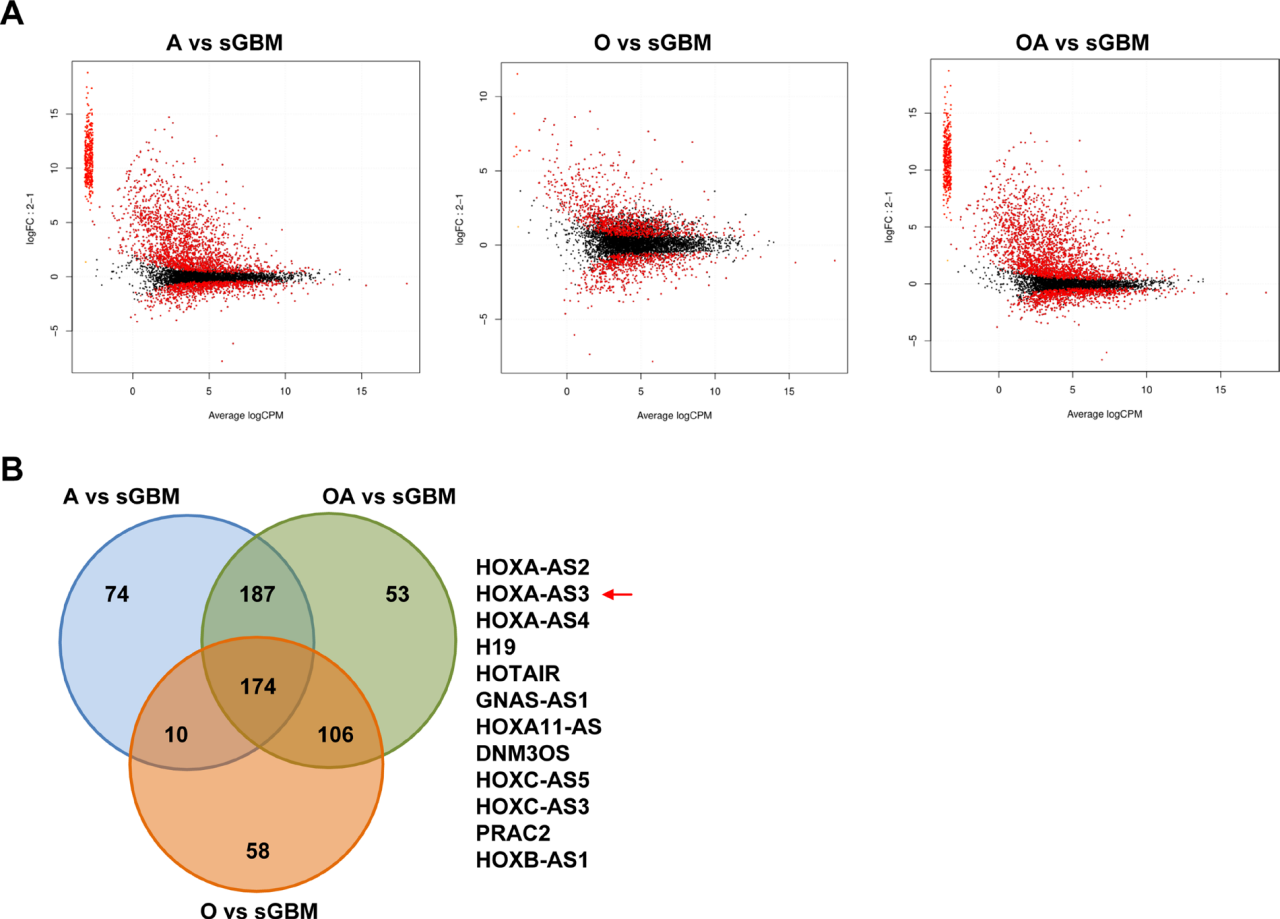
Differentially expressed (DE) ncRNAs between grade II gliomas and sGBM (**A**) plotSmear showing the DE ncRNA between grade II gliomas and sGBM. A: astrocytoma; O: oligodendroglioma; OA: oligoastrocytoma. The red spot represents the DE ncRNAs (**B**) Venn diagram depicting the overlapped ncRNAs between the three different comparisons.

To investigate the role of lncRNAs in glioma progression, the RNAseq data form CGGA cohort was used to analyze the differentially expressed lncRNAs. First, we profiled the expression of ncRNAs and obtained 732 annotated ncRNAs in the CGGA cohort by using the method [18]. Since the low grade gliomas will develop into secondary glioblastoma (sGBM) partially, SAM analysis with edgeR package (*P* value < 0.05, FDR < 0.05) was performed to compare the differentially expressed ncRNAs between grade II gliomas (35 astrocytoma, 27 oligodendroglioma, 33 oligoastrocytoma) and sGBM (34) respectively (Figure 1A). 174 ncRNAs of aberrant expression were observed, and 12 up-regulated ncRNAs of most significance were listed in Figure 1B, including several tumorigenesis-related lncRNA (H19, HOTAIR).

**Figure 2:**
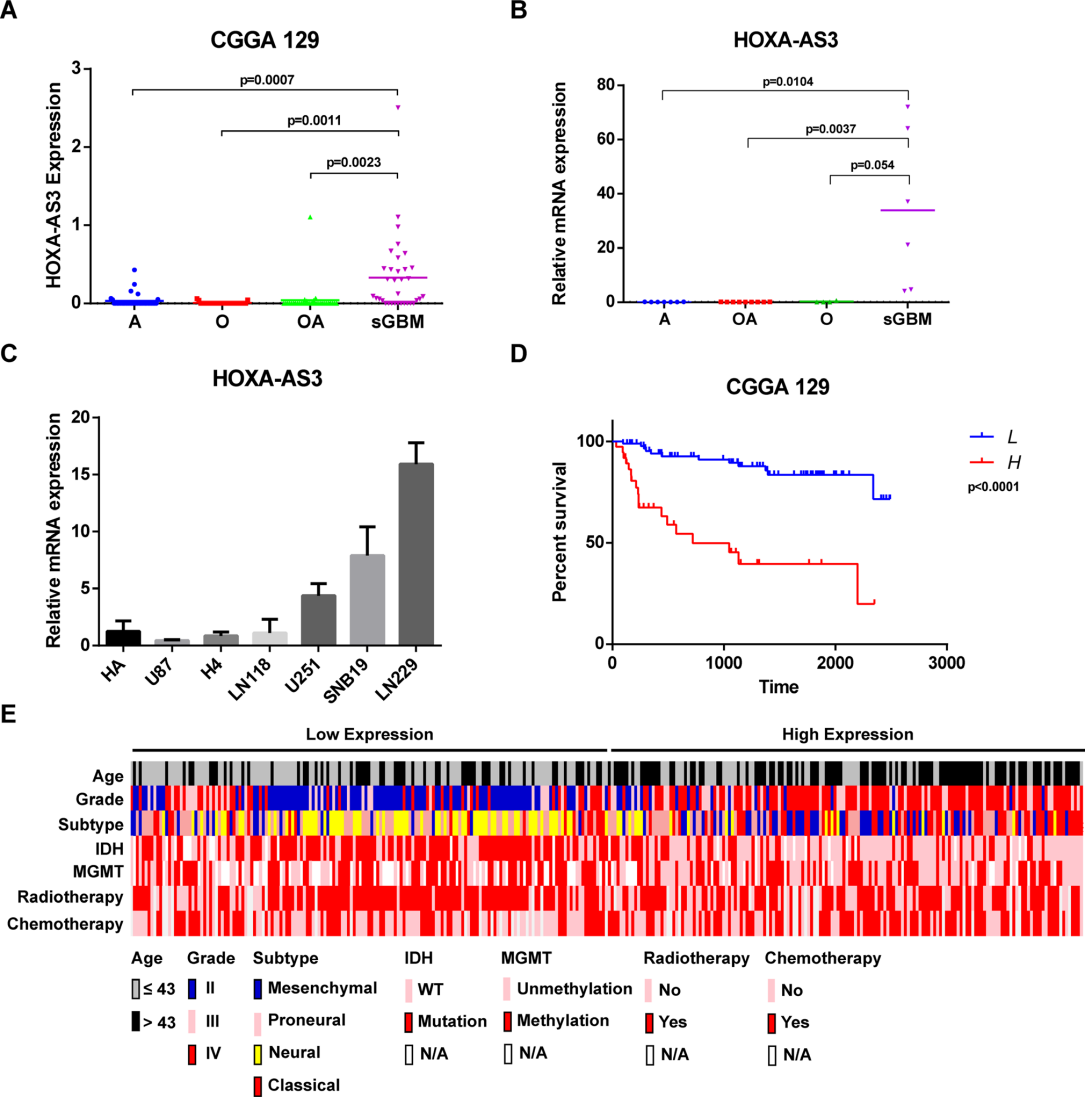
HOXA-AS3 expression and clinical significance in gliomas (**A**) HOXA-AS3 expression analysis in glioma patients by using the RNAseq data form CGGA cohort (129 glioma cases). (**B**) qPCR analysis of relative HOXA-AS3 expression in 26 glioma samples (7 astrocytoma, 9 oligodendroglioma, 4 oligoastrocytoma and 6 sGBM). Bars represent median HOXA-AS3 level. (**C**) qPCR analysis of relative HOXA-AS3 expression in normal glial cell and glioma cell lines. (**D**) Kaplan-Meier analysis of overall survival based on HOXA-AS3 level in 129 cases of glioma patients. Glioma patients were divided into HOXA-AS3 high expression group and low expression group. (**E**) Distribution of clinical and molecular pathological features according to HOXA-AS3 expression in 325 glioma patients.

Among these up-regulated ncRNAs, The RNAseq data showed that lncRNA HOXA-AS3 expression level was evaluated in sGBM samples than in grade II glioma samples (Figure 2A). After that, we performed quantitative Real-time PCR analysis on tumor samples from sGBM and grade II glioma patients. Consistent with the RNAseq data, the level of HOXA-AS3 was significantly increased in sGBM samples (Figure 2B). We next used CGGA as another larger dataset (*n* = 325 glioma samples) to validate the expression of HOXA-AS3. We found that lncRNA HOXA-AS3 expression was gradually increased along with glioma pathological grade (Supplementary Figure 1A). To validate the RNAseq data, HOXA-AS3 expression level was determined in an another cohort of 47 glioma samples (containing 24 grade II, 12 grade III and 11 grade IV cases) by quantitative Real-time PCR. The results revealed that HOXA-AS3 expression was upregulated (*P* < 0.05) in grade IV glioma tissues compared with grade II and III tissues (Supplementary Figure 1B). Moreover, as shown in Supplementary Figure 1C, HOXA-AS3 was highly expressed in GBM samples compared with normal brain tissues. HOXA-AS3 expression was also detected in the glioma cell lines, including U87, H4, LN118, U251, SNB19 and LN229, and the normal glial cell line HA. Significantly high HOXA-AS3 expression was found in U251, SNB19 and LN229 (*P* < 0.05) compared with that in HA (Figure 2C). These findings provided that lncRNA HOXA-AS3 was increased in expression in high grade of gliomas.

### High HOXA-AS3 expression is associated with poor prognosis in glioma patients

Kaplan-Meier survival analysis with a log-rank comparison was conducted to investigate the correlation between HOXA-AS3 expression and overall survival of glioma patients. 129 glioma patients were classified into two groups: the high HOXA-AS3 group (*n* = 90, expression higher than median level) and the low HOXA-AS3 group (*n* = 39, expression lower than median level) according to relative HOXA-AS3 expression. Patients with high HOXA-AS3 expression have shorter overall survival than those with low expression of HOXA-AS3 (Figure 2D). The similar result was observed in the larger CGGA cohort (*n* = 325) (Supplementary Figure 1E). Furthermore, HOXA-AS3 expression was inversely correlated with overall survival in grade II (*P* = 0.0066, Supplementary Figure 1F) and grade III (*P* = 0.003, Supplementary Figure 1G) samples, but not in grade IV samples (*P* = 0.0875, Supplementary Figure 1H). The relationship between HOXA-AS3 expression and clinicopathologic factors was also analyzed (Figure 2E). As shown in Table 1, increased HOXA-AS3 levels were correlated with age (*P* < 0.001), subtype (*P* < 0.001), grade (*P* < 0.001), IDH1 mutation (*P* < 0.001), MGMT promoter methylation (*P* = 0.031), radiotherapy (*P* = 0.043) and chemotherapy (*P* = 0.006). However, there was no significant correlation between HOXA-AS3 expression and gender (*P* > 0.05). Univariate regression analysis found that HOXA-AS3 expression, age, subtype, grade, IDH1 mutation, MGMT promoter methylation and radiotherapy were potential survival predictors (Table 2). Further analysis in multivariate Cox regression showed that HR for HOXA-AS3 expression is 1.735 (95% CI: 1.073–2.806, *P* = 0.025) of overall survival, indicating that HOXA-AS3 expression was a potential independent prognostic factor in glioma.

**Table 1:** Correlation between HOXA-AS3 expression and clinicopathologic factors of glioma patients

Characteristics	*n*	HOXA-AS3 expression		*P*-value
		Low	High	
**Total Cases**	325	163	162	
**Age**				
≤ 43	163	103	60	**< 0.001**
> 43	162	60	102	
**Gender**				
Male	203	100	103	0.678
Female	122	63	59	
**Subtype**				
Classical	74	20	54	**< 0.001**
Mesenchymal	68	21	47	
Proneural	102	59	43	
Neural	81	63	18	
**Grade**				
II	109	90	19	**< 0.001**
III	72	32	40	
IV	144	41	103	
**IDH1 mutation**				
MUT	152	104	48	**< 0.001**
WT	148	47	101	
NA	25	12	13	
**MGMT promoter methylation**				
Methylation	139	71	68	**0.031**
Unmethylation	117	44	73	
NA	69	48	21	
**Radiotherapy**				
Yes	212	116	96	**0.043**
No	84	35	49	
NA	29	12	17	
**Chemotherapy**				
Yes	158	67	91	**0.006**
No	128	75	53	
NA	39	21	18	

**Table 2:** Univariate and multivariate analysis of survival in glioma patients

Characteristics	Univariate analysis			Multivariate analysis		
	HR	95% CI	*P*-value	HR	95% CI	*P*-value
**Age**	1.888	1.347 – 2.648	**< 0.001**	0.932	0.576 – 1.507	0.774
**Gender**	1.181	0.837 – 1.666	**0.345**			
**Subtype**	0.551	0.473 – 0.642	**< 0.001**	0.81	0.657 – 0.998	**0.048**
**Grade**	3.477	2.716 – 4.452	**< 0.001**	2.232	1.6 – 3.114	**< 0.001**
**IDH1 mutation**	0.253	0.172 – 0.37	**< 0.001**	0.617	0.343 – 1.11	0.107
**MGMT promoter methylation**	0.526	0.371 – 0.745	**< 0.001**	0.721	0.472 – 1.101	0.13
**Radiotherapy**	0.429	0.296 – 0.622	**< 0.001**	0.483	0.316 – 0.738	**0.001**
**Chemotherapy**	1.378	0.963 – 1.971	**0.079**			
**HOXA-AS3**	4.126	2.865 – 5.942	**< 0.001**	1.735	1.073 – 2.806	**0.025**

Age: ≤ 43, > 43; Gender: Male, Female; Subtype: Classical, Mesenchymal, Proneural, Neural; Grade: II, III, IV; IDH1 mutation: MUT, WT; MGMT promoter methylation: Methylation, Unmethylation; Radiotherapy: Yes, No; Chemotherapy: Yes, No; HOXA-AS3: Low expression, High expression.

### Effects of HOXA-AS3 on the proliferation of glioma cells

**Figure 3:**
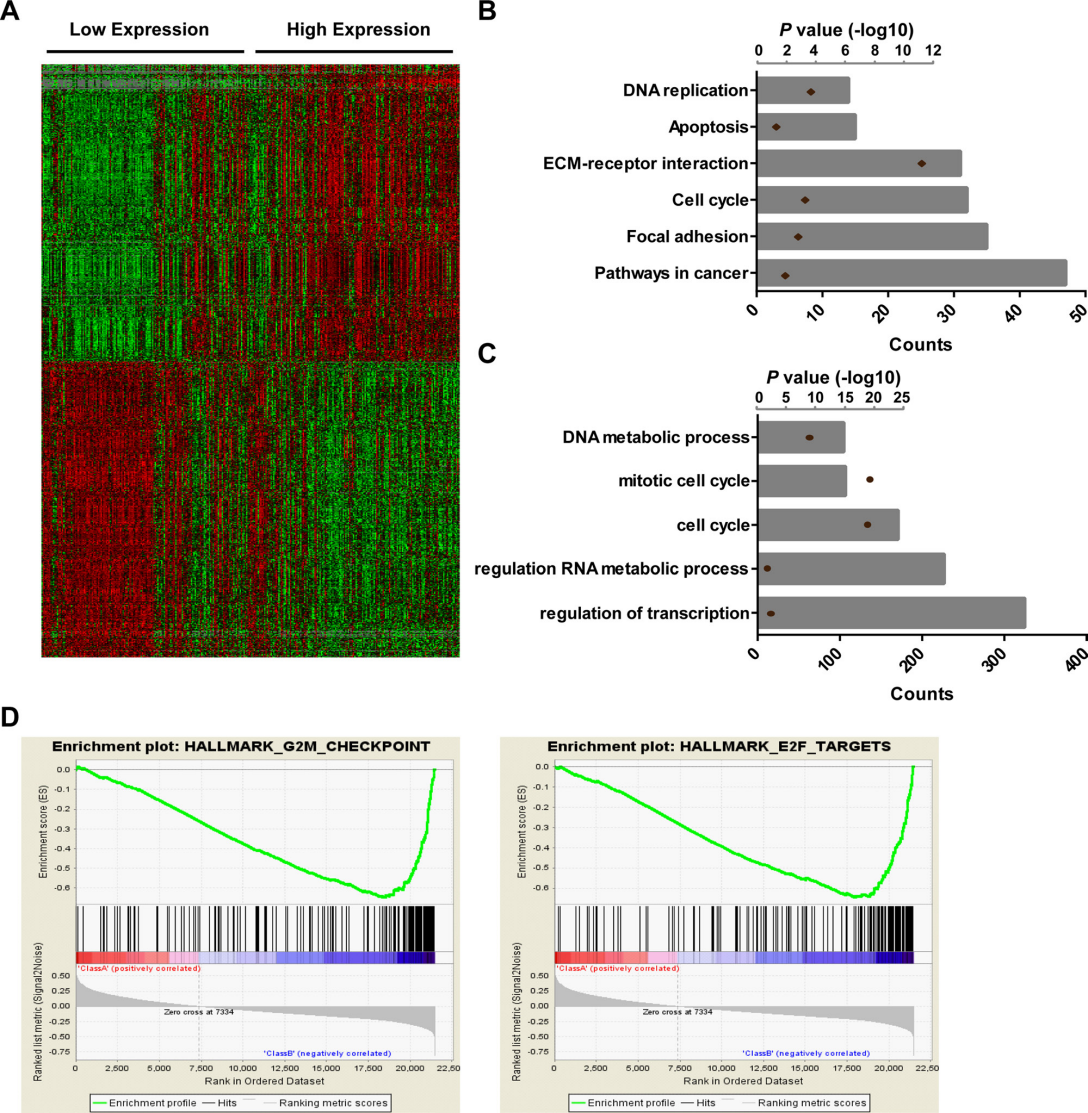
Biological function analysis of HOXA-AS3 in gliomas (**A**) SAM was performed to identify genes correlated with HOXA-AS3 expression using R software (False Discovery Rate, FDR < 0.01). Heat map was constructed using Gene Cluster and Tree View software with the top 500 genes positively and negatively correlated with HOXA-AS3 expression. (**B**) KEGG pathway analysis by using HOXA-AS3 positively associated genes. (**C**) GO biological processes analysis by using HOXA-AS3 positively associated genes. Column height: gene counts; point height: *P* value. (**D**) GSEA analysis based on the median of HOXA-AS3 expression levels.

To elucidate whether HOXA-AS3 plays a role in glioma tumorigenesis, SAM analysis was performed to cluster genes in the RNAseq data from CGGA according to the expression of HOXA-AS3. The top 500 genes positively and negatively associated with HOXA-AS3 were showed in the heatmap (Figure 3A), and the gene list was showing in Supplementary Table 2. KEGG pathway analysis revealed that HOXA-AS3 is highly correlated with genes involved in pathways in cancer, focal adhesion, cell cycle, ECM-receptor interaction, apoptosis and DNA replication (Figure 3B). Furthermore, gene ontology analysis found that the most representative biological processes were regulation of transcription, regulation of RNA metabolic process, cell cycle, mitotic cell cycle and DAN metabolic process (Figure 3C). GSEA analysis was also performed based on the median of HOXA-AS3 expression levels. Enrichment plots of GSEA showed that the gene signatures of G2M checkpoint and E2F targets were enriched in patients with high HOXA-AS3 expression (Figure 3D). These data suggest that HOXA-AS3 may be a crucial modulator in glioma tumorigenesis.

**Figure 4:**
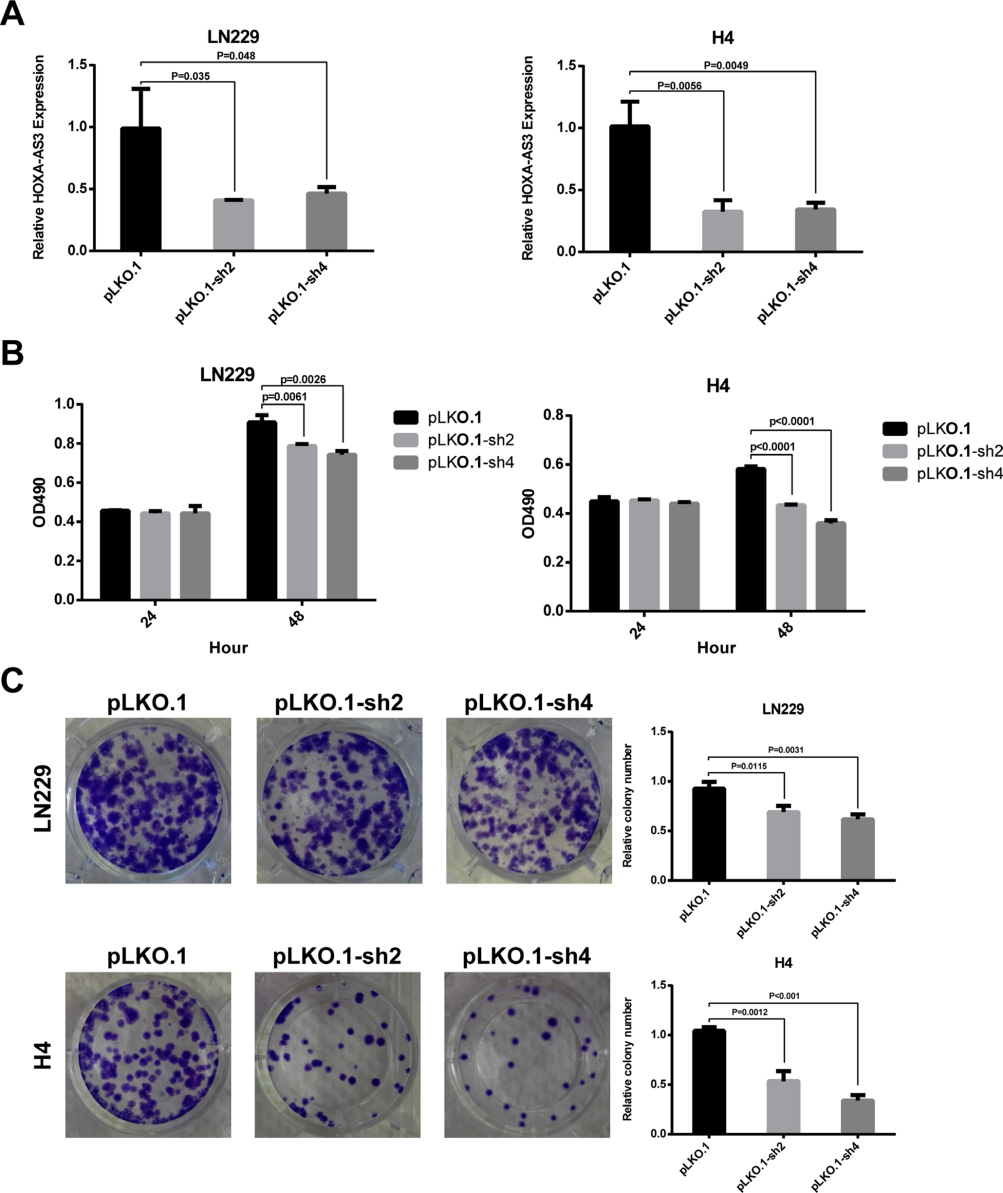
Knockdown of HOXA-AS3 inhibits glioma cell growth *in vitro* (**A**) qPCR analysis of the knockdown efficiency of sh2 and sh4 in LN229 and H4 cells. (**B**) MTS assay was performed to determine the cell viability after transfection with pLKO.1 or pLKO.1-shRNAs. (**C**) Colony formation assay was conducted to determine the effect of HOXA-AS3 knockdown on cloning ability in LN229 and H4 cells. Data were shown as mean ± SD.

To functionally validate the effects of HOXA-AS3 on glioma cell phenotype, shRNAs were transfected to knock down endogenous HOXA-AS3 in glioma cell lines. To exclude off-target effect, two different shRNAs (sh2 and sh4) were designed. Results of qPCR analysis showed that both of them could efficiently knock down the HOXA-AS3 level in LN229, H4 (Figure 4A) and SNB19 cells (Supplementary Figure 2A). MTS assay revealed that cells transiently transfected with sh2 and sh4 had inhibited growth compared with controls (Figures 4B and Supplementary Figure 2B). Consistent with the results of MTS assays, colony formation assay showed that clonogenic ability was impaired following knockdown of HOXA-AS3 in LN229 and H4 cells (Figure 4C). In addition, to determinate whether HOXA-AS3 inhibits the growth of glioma cell specifically, we performed knockdown assay in two normal glial cells (HA and HAC). As shown in Supplementary Figure 3, HOXA-AS3 knockdown did not suppress the growth of normal glial cells. These results provide evidence of the growth-promoting role of HOXA-AS3 in glioma cells.

### HOXA-AS3 regulates cell-cycle progression, apoptosis and migration of glioma cell

**Figure 5:**
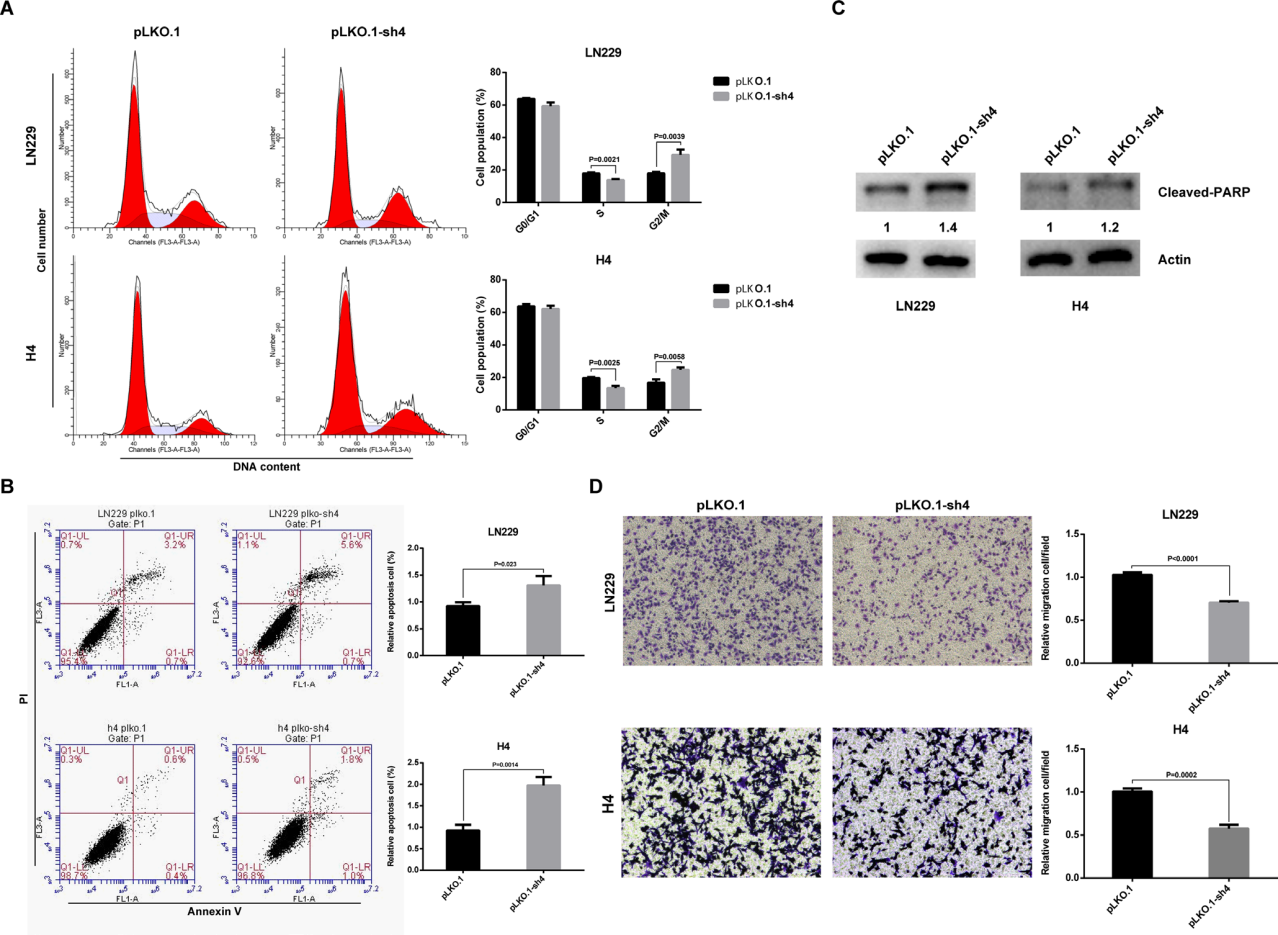
Effects of HOXA-AS3 on glioma cell cycle progression, apoptosis and migration *in vitro* (**A**) LN229 and H4 cells were treated with pLKO.1 or pLKO.1-sh4 and subjected to cell cycle analysis by flow cytometry. (**B**) Flow cytometry assays were performed to determine the cell apoptosis in pLKO.1-sh4-transfected LN229 and H4 cells. (**C**) Immunoblot analysis of apoptosis marker (cleaved-PARP) in cells transfected with pLKO.1 (control) or pLKO.1-sh4. The numbers represent gray value relative to Actin. (**D**) Transwell assays were conducted to assess the effect of HOXA-AS3 knockdown on cell migration. Data were shown as mean ± SD.

To determine whether the effects of HOXA-AS3 on glioma cell proliferation were influenced by cell cycle arrest and apoptosis, flow cytometric analysis was performed. The results showed that glioma cells transfected with shRNA had a cell cycle arrest at the G2/M phase and the percentage of S-phase cells decreased (Figure 5A). Annexin V and PI staining revealed that the fraction of apoptotic cells following shRNA treatment was increased relative to that in control groups (Figure 5B). Furthermore, western blot analysis indicated that the cleaved-PARP level was also increased in the shRNA treated LN229 and H4 cells (Figure 5C). These data imply that G2/M arrest and increased apoptosis may contribute to the growth inhibition mediated by HOXA-AS3 knockdown. To investigate whether HOXA-AS3 is involved in the progression of glioma, the effect of HOXA-AS3 on migration ability was detected by using a transwell system. The results indicated that HOXA-AS3 knockdown significantly impeded the migration when compared with control cells (Figures 5D and Supplementary Figure 2C). Taken together, these results suggest that HOXA-AS3 plays important roles in glioma malignant phenotypes.

### Knockdown of HOXA-AS3 inhibits glioma cells tumorigenesis *in vivo*

**Figure 6:**
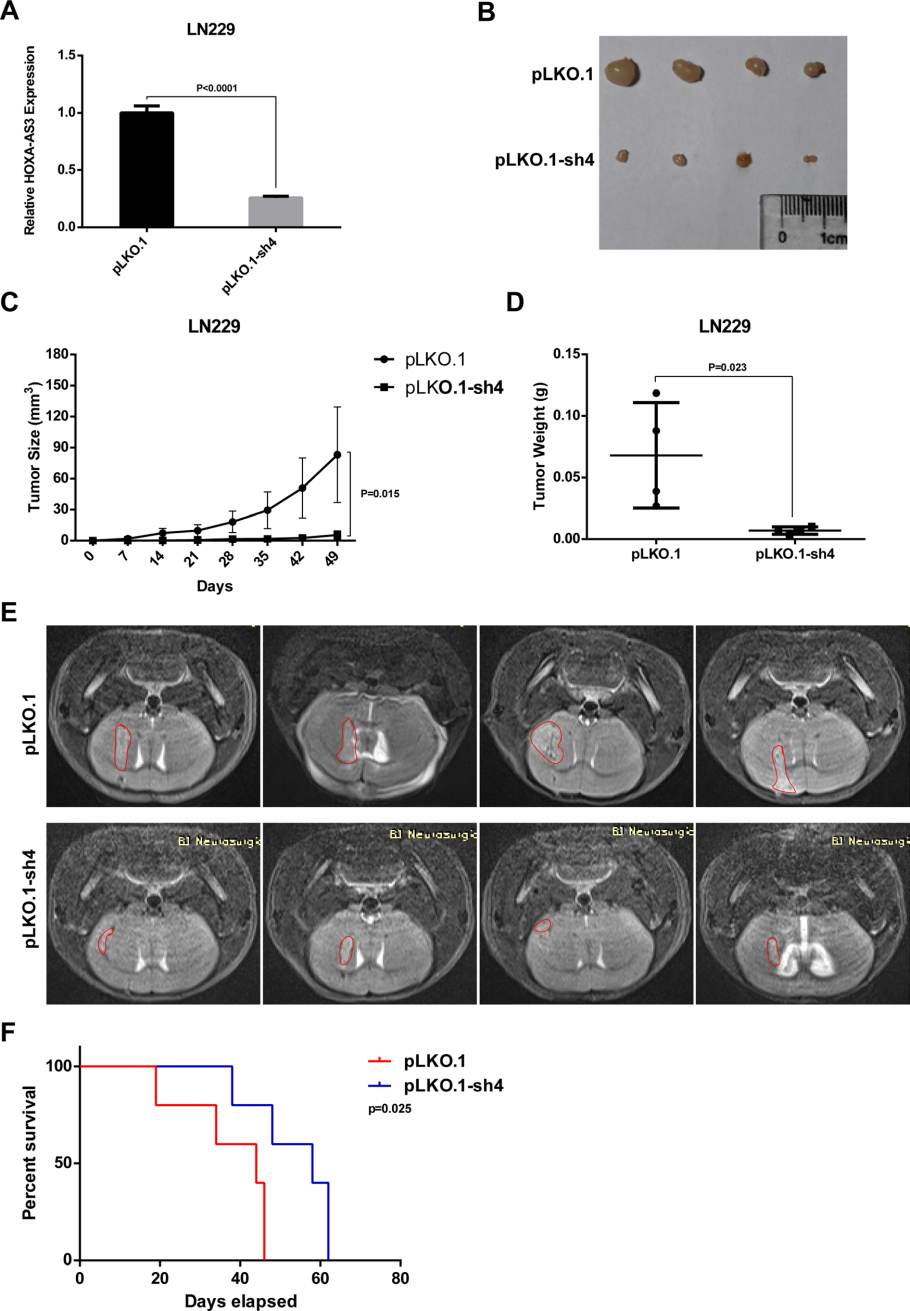
Knockdown of HOXA-AS3 inhibits glioma cell growth *in vivo* (**A**) qPCR analysis of the knockdown efficiency of pLKO.1-sh4 in the stable HOXA-AS3 knockdown LN229 cells. (**B**) Tumors isolated form nude mice of pLKO.1 and pLKO.1-sh4 group (*n* = 4). (**C**, **D**) Tumor size and weight of each tumor sample form two groups are represented. (**E**) MRI analysis of the intracranial tumors from pLKO.1 (*n* = 4) and pLKO.1-sh4 (*n* = 4) groups. (**F**) Kaplan-Meier analysis of overall survival in mice injected with pLKO.1 or pLKO.1-sh4 LN229 cells. *n* = 5, Log-rank test. Data were shown as mean ± SD.

To confirm the impact of HOXA-AS3 on glioma cells growth *in vivo*, LN229 cells were stably infected with pLKO.1 or pLKO.1-sh4 lentivirus. The qPCR analysis showed HOXA-AS3 expression was knocked down by 75% in pLKO.1-sh4 lentivirus –infected group (Figure 6A). Then, cells were subcutaneously injected in the right frank of nude mice. The tumors formed in the pLKO.1-sh4 group were obviously smaller than those in the control group (Figure 6B and 6C). Meanwhile, the tumor weight was significantly lower in the pLKO.1-sh4 group relative to those of the control group (Figure 6D). Furthermore, the intracranial model was also used and cells were orthotopically injected into nude mice. MRI found that the intracranial tumors grew much slower when HOXA-AS3 was knocked down (Figure 6E). Kaplan-Meier analysis showed that the overall survival time of xenograft mice was much longer in the pLKO.1-sh4 group (Figure 6F). Collectively, these results complement the above *in vitro* studies and demonstrate that HOXA-AS3 knockdown inhibits tumor growth *in vivo*.

## DISCUSSION

Over the past decades, significant developments of sequencing technique and bioinformatics had been made. Hundreds of lncRNAs were identified and their aberrant expression has been associated with tumor development and progression in various types of cancers [19]. Recently, to uncover the expression pattern of lncRNAs in glioma, high throughput microarrays have been used to profile lncRNAs expression in different grades and histological subtypes of gliomas. Kang et al. found that 1308 lncRNAs were differentially expressed between glioblastoma multiforme (GBM) and normal brain tissues by using microarray platform, ASLNC22381 and ASLNC2081 play roles in the regulation of glioma signaling pathways [20]. Using a microarray-mining approach, Zhang et al. identified 127 lncRNAs that were differentially expressed between gliomas and non-tumoral brain tissues [21]. In the present study, the RNAseq data form CGGA including 325 glioma tissues with different WHO grades was analyzed to identify differentially expressed lncRNAs. We found a new lncRNA HOXA-AS3 and demonstrated that its expression was upregulated in high grade gliomas. Increased HOXA-AS3 expression was further verified in glioma samples of different grades by qPCR. In addition, we analyzed the HOXA-AS3 expression in four GBM subtypes according to The Cancer Genome Atlas (TCGA) GBM molecular classification. Interestingly, HOXA-AS3 expression in the classical subtype was higher than that in the mesenchymal, neural and proneural subtypes (Supplementary Figure 1D). lncRNA HOXA-AS3 is located in chromosome 7p15.2. Since gain of chromosome 7 is one of most common genetic alteration in glioblastoma [22], the increased expression of HOXA-AS3 might be attributed to the amplification of chromosome 7, or the regulation of transcriptional activation and mRNA stability, and the amplification of chromosome 7 may be the main reason of upregulation of HOXA-AS3 which needs further study. Moreover, Kaplan-Meier analysis revealed that high HOXA-AS3 expression is associated with poor prognosis in glioma patients. Univariate and multivariate analysis indicated that HOXA-AS3 expression provided a significantly independent prognostic maker in glioma. In addition, we also investigated whether HOXA-AS3 affects IDH1 mutation and MGMT promoter methylation in glioma cells. In order to detect whether HOXA-AS3 affects MGMT promoter methylation, we performed pyrosequencing with stable LN229 cell lines (pLKO.1, pLKO.1-sh2 and pLKO.1-sh4) using MGMT Pyro Kit (Qiagen). The result found the knockdown groups (pLKO.1-sh2 and pLKO.1-sh4) show the similar methylation level in 4 CpG sites in exon 1 of MGMT gene with the control group. After sequencing, the IDH1 mutation (R132H) of knockdown group also has no significant change compared with control group (Supplementary Figure 4). Although the increased HOXA-AS3 levels were correlated with MGMT promoter methylation and IDH1 mutation, the sequencing data indicate that HOXA-AS3 might have no effect on MGMT promoter methylation and IDH1 mutation.

HOXA-AS3 is located on the antisense strand of HOXA gene clusters in chromosome 7. The homebox (HOX) gene family consisting of 39 genes plays a major role in tissue development but also in cancers [23, 24]. Near the HOX genes are many famous antisense transcripts which are involved in cancer development and progression. For example, HOTAIR regulates polycomb-dependent chromatin modification and is associated with poor prognosis in colorectal cancers [25]. In pancreatic cancer, the long non-coding RNA HOTTIP promotes progression and gemcitabine resistance by regulating HOXA13 [26]. Long intergenic non-coding RNA HOTAIRM1 regulates cell cycle progression during myeloid maturation in leukemia cells [27]. Wang et al. reported that HOXA11-AS is a novel cell cycle-associated lncRNA and a biomarker of progression in glioma [18]. lncRNA HOXA-AS2 promotes gastric cancer proliferation by epigenetically silencing P21/PLK3/DDIT3 expression [28]. Our studies revealed that HOXA-AS3 knockdown inhibited the proliferation through impeding cell cycle progression and promoting cell apoptosis, and impaired cell migration in glioma cells. Moreover, knockdown of HOXA-AS3 inhibited glioma cells tumorigenesis *in vivo*. These findings indicate that HOXA-AS3 may function as an oncogene and its high expression contributes to glioma progression.

Recently, it became obvious that lncRNAs play an important role in regulating target genes through various mechanisms. lncRNAs can recruit chromatin-modifying enzymes to target genes and regulate their transcription either in *cis* or in *trans*. For example, lncRNA AGAP2-AS1 represses LATS2 and KLF2 expression through interacting with EZH2 and LSD1 in non-small-cell lung cancer cells [29]. Some lncRNAs can serve as molecular scaffolds to bind relevant components to regulate gene expression. For instance, lncRNA GClnc1 promotes gastric carcinogenesis by acting as a modular scaffold of WDR5 and KAT2A complexes to specify the histone modification pattern [30]. Some lncRNAs can also act as a molecular decoy. In gastric cancer, lncRNA TINCR regulates cell proliferation and apoptosis through binding to STAU1 to affect KLF mRNA stability [31]. In addition, lncRNAs can function as competing endogenous RNAs (ceRNAs) by binding with miRNA. For example, knockdown lncRNA XIST exerts tumor suppressive functions by up-regulating miR-152 in human glioblastoma stem cells [32]. In our studies, KEGG and GO analysis revealed a strong association between HOXA-AS3 and transcription regulation, which implies that HOXA-AS3 may exert its important role in glioma through transcription regulation. In addition, GSEA analysis revealed that the gene signatures of G2/M checkpoint and E2F targets were enriched in patients with high HOXA-AS3 expression. The E2F transcription factor family is known to play a crucial role in cell cycle progression. It's reported that E2F4 targets contain genes involved in G2/M checkpoint. The promoters of G2/M checkpoint genes chk1 and mad2 were bound by E2F4 transcription factor [33]. Therefore, HOXA-AS3 might affect cell growth by regulating the G2/M checkpoint genes chk1 and mad2 through interacting with E2F4. The mechanism by which HOXA-AS3 promotes glioma cell growth will be verified in the next work.

In summary, our study had shown that HOXA-AS3 was upregulated in glioma tissues and cell lines and its high expression was associated with poor prognosis of glioma patients for the first time. Knockdown of HOXA-AS3 exerted tumor-suppressive functions through inhibiting cell proliferation and inducing apoptosis in glioma cells. *In vivo* assay further confirmed the growth-inhibiting role of HOXA-AS3 knockdown. Our findings provide a new perspective that HOXA-AS3 may acts as an oncogene in glioma progression and a new target for treatment of glioma. However, further studies are required to clarify the possible mechanism by which HOXA-AS3 regulates glioma cell biological function.

## MATERIALS AND METHODS

### Clinical specimens and cell lines

325 glioma samples (consisting of 109 grade II, 72 grade III and 144 grade IV samples) from the Chinese Glioma Genome Atlas (CGGA) were included in this study. An additional 47 frozen glioma samples were obtained from the glioma center of Beijing Tiantan hospital. All these samples were histologically diagnosed according to the 2007 World Health Organization (WHO) classification of tumors of the nervous systems. The study protocol was approved by the ethics committee of Tiantan hospital. Written informed consents were obtained from all participants in this study. Human glioma cell lines LN229, U251, SNB19, U87 and H4 were purchased from the American Type Culture Collection (ATCC, USA), LN118 and normal glial cell lines HA and HAC were kindly provided by Prof. Peng of School of Basic Medicine Peking Union Medical College (Bejing, China). All the glioma cell lines cultured in Dulbecco’s-modified Eagle medium (DMEM) F12 with 10% fetal bovine serum (FBS, Gibco). Normal glial cell lines were cultured in Astrocyte Medium (AM).

### Quantitative real-time PCR

Total RNA was extracted from glioma specimens or cell lines using Trizol reagent (Life Technologies). cDNA was synthesized using RevertAid First Strand cDNA Synthesis Kit (Thermo Scientific) according to the manufacturer's protocol. Quantitative real-time PCR was performed using the standard protocol from the SYBR Select Master Mix kit on Applied Biosystems 7500 real-time system (Applied Biosystems, USA). Each sample was analyzed in triplicate. GAPDH was used as the internal standard and the primer sequences were showed in Supplementary Table 1.

### MTS and Colony formation assay

Cell viability was assessed using a CellTiter 96 Non-Radioactive Cell Proliferation Assay kit (Promega, USA) according to the manufacturer's instructions. LN229 and H4 cells were plated in 96-well plates (2 × 10^3^ cells/well). After 24 hours, cells were transfected with shRNA plasmids and then cultured for 1 and 2 day. The medium in each well was replaced with 120 μl of complete medium containing 20 μl of MTS (3-(4,5-dimethylthiazol-2-yl)-5-(3-carboxymethoxyphenyl)-2-(4-sulfophenyl)-2H-tertrazolium, inner salt) solution, and the plates were incubated for 1 hour at 37°C. The absorbance (490 nm) was measured at the indicated time points. For colony formation assay, 24 hours post-transfection, cells were seeded into 12-well plates (10^2^ cells/well) and cultured for 8–14 days. The cells were stained with 0.1% crystal violet at the end of time course and the number of colonies was counted.

### Flow cytometry

Cell cycle and apoptosis were analyzed by flow cytometry. For cell cycle analyses, LN229 and H4 cells were harvested 2 d after transfection with shRNA plasmids, wash once with PBS, and fixed in 70% ethanol. After incubating with RNase A for 30 min at 37°C, cells were labeled with propidium iodide (Sigma-Aldrich, USA) and analyzed on Accuri C6 flow cytometer (DB Pharmigen, USA). The cell phase was analyzed by ModFit LT software. The late and early apoptotic cells were measured using the FITC Annexin V Apoptosis Detection Kit I (BD Pharmigen, USA) by Accuri C6 flow cytometer analysis 72 hours after transfection.

### Cell migration assay

Cell migration assay was performed using 24-well transwell chambers with 8 μm pore (Corning, USA). Cells were harvested 24 h after transfection with shRNA plasmids. 1 × 10^5^ cells were seeded into the upper chamber in 100 μl of serum-free medium. 500 μl of medium containing 20% serum was added to the lower chamber as the chemoattractant. After 3–4 h incubation, the migrated cells at the bottom surface were fixed and stained with 0.5% crystal violet. 6 random fields in each well were counted.

### *In vivo* assay

All protocols were approved by the Institutional Animal Care and Use Committee at Beijing Neurosurgical institute, Capital Medical University. 4 or 6-week-old female BALB/c nude mice were used for subcutaneous and intracranial model. LN229 cells were stably infected with pLKO.1 or pLKO.1-sh4 lentivirus, harvested and washed with PBS. 5 × 10^5^ cells in 100 μl PBS were subcutaneously injected in the right frank of each mouse. Tumor growth was measured every 7 days, and the tumor volume was calculated using the equation V = L × W^2^ × π/6 (V, volume; L, length; W, width). For the orthotopic model, 5 × 10^5^ cells in 5 μl PBS were injected into the right striatum of each mouse (*n* = 4). In order to monitor the tumor size between pLKO.1 or pLKO.1-sh4 groups, small animal magnetic resonance imaging (MRI) (Bruker Biospec, 7.0T) was performed using resting-state functional MRI (re-fMRI) approach before mice died. Before performing MRI analysis, each mouse was anesthetized by using 0.7% pentobarbital sodium. For the Kaplan-Meier analysis of overall survival, another orthotopic assay was proceeded. pLKO.1 or pLKO.1-sh4 cells were injected into the right striatum of each mouse (*n* = 5). Then, the time of death of each mouse was recorded for Kaplan-Meier analysis.

### Plasmid construction, lentivirus production and cell infection

HOXA-AS3-sh2 and sh4 (sequences were listed in Supplementary Table 1) were cloned in the lentiviral vector pLKO.1. The positive clones were verified by DNA sequencing. The correct plasmids were cotransfected with the packaging vectors pLP1, pLP2 and pLP/VSVG into 293T cells using X-tremeGENE HP DNA transfection reagent (Sigma-Aldrich, USA). Virus supernatants were collected and purified by ultracentrifugation. For infection, LN229 cells were then maintained in the medium containing pLKO.1/pLKO.1-sh4 lentivirus. After 48 hours, the stable line was selected by puromycin (1.5 μg/ml) and used for the *in vivo* assay.

### Western blot analysis

Cells transfected with pLKO.1 or pLKO.1-sh4 were harvested and lysed for protein collection. Protein samples were separated by SDS-PAGE and transferred onto PVDF membrane. Then, the membrane was immunostained with the antibody according to standard protocols. Antibodies used in this study were as followed: cleaved-PARP (Abcam) and Actin (Santa Cruz).

### Statistical analysis

All data were presented as mean ± standard error, two-sided *P*-value < 0.05 was considered stastistically significant. The Student's *t*-test and *x*^2^ test were used for comparisons between groups. The overall survival rates were calculated by Kaplan-Meier method with log-rank test, using GraphPad Prism 5.0. Univariate and multivariate Cox regression analysis were performed to test for independed prognostic factors in SPSS13.0. The Gene Set Enrichment Analysis (GSEA) was performed to determine functional gene sets using Gene Set Enrichment software. Heat map was constructed using Gene Cluster 3.0 and Tree View software. The significance analysis of microarray (SAM) was calculated using R software 3.2.3 with the samr package (False Discovery Rate, FDR < 0.01).

## SUPPLEMENTARY MATERIALS FIGURES AND TABLE




