# Supplementary material for: Upregulation of long noncoding RNA HOXA-AS3 promotes tumor progression and predicts poor prognosis in glioma
Source: Oncotarget. 2017 May 24;8(32):53110–23. doi: 10.18632/oncotarget.18162 (PMC5581096; doi:10.18632/oncotarget.18162)
Supplement: Supplementary file 1 [file oncotarget-08-53110-s001.pdf]

Upregulation of long noncoding RNA HOXA-AS3 promotes tumor progression and predicts poor prognosis in glioma

Supplementary Materials

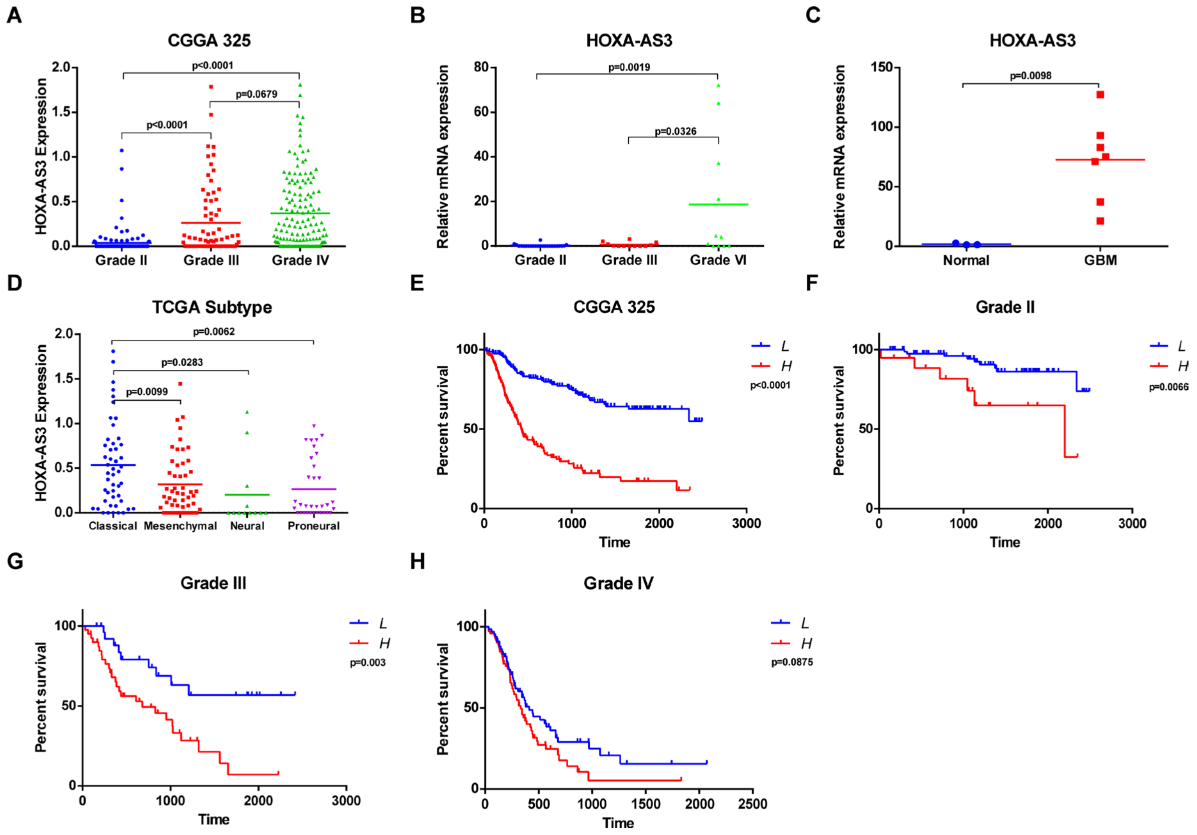

Supplementary Figure 1: HOXA-AS3 expression and clinical significance in CGGA cohort (n = 325). (**A**) HOXA-AS3 expression analysis in glioma patients by using the RNAseq data form CGGA cohort (325 glioma cases). (**B**) qPCR analysis of relative HOXA-AS3 expression in 47 glioma samples. Bars represent median HOXA-AS3 level. (**C**) qPCR analysis of relative HOXA-AS3 expression in normal brain tissues (n = 3) and GBM samples (n = 7). (**D**) HOXA-AS3 expression analysis between the four GBM subtypes in CGGA dataset. (**E–H**) Kaplan-Meier analysis of overall survival based on HOXA-AS3 level in 325 cases of glioma patients, grade II, grade III and grade IV patients. Glioma patients were divided into HOXA-AS3 high expression group and low expression group.

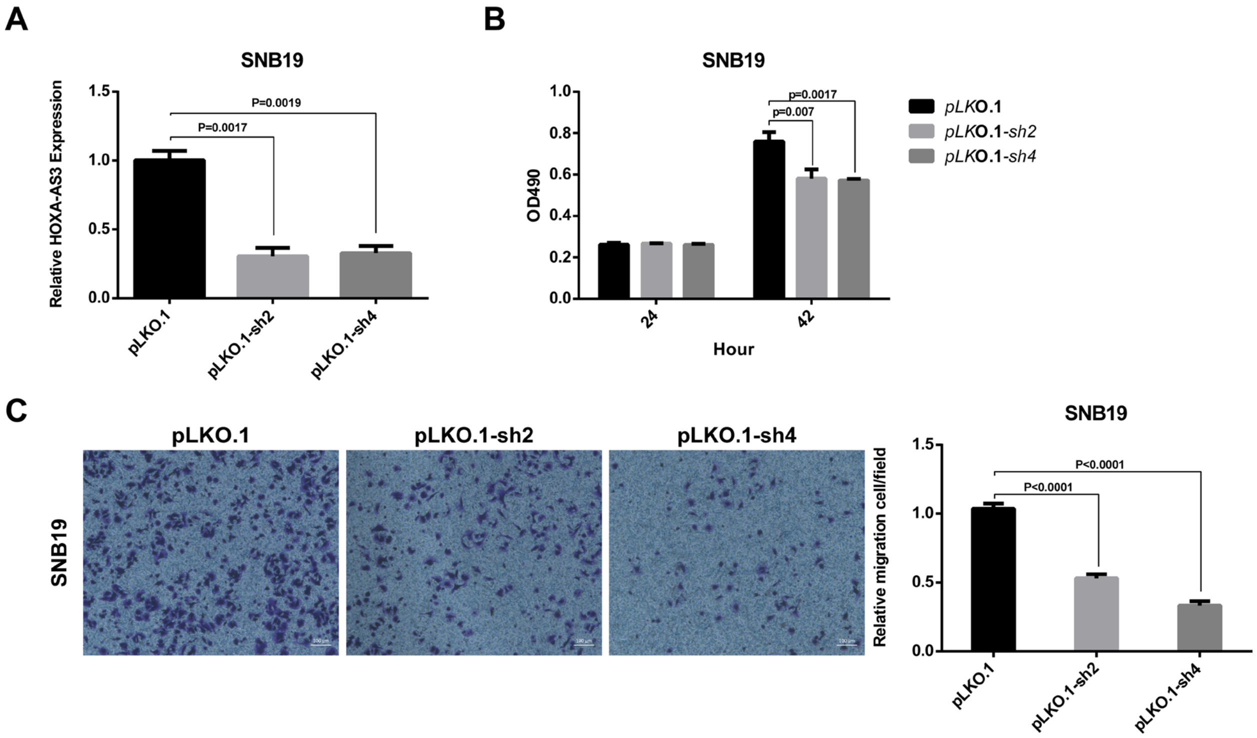

Supplementary Figure 2: Knockdown of HOXA-AS3 inhibits glioma cell growth and migration in SNB19 cell. (**A**) qPCR analysis of the knockdown efficiency of sh2 and sh4 in SNB19 cells. (**B**) MTS assay was performed to determine the cell viability after transfection with pLKO.1 or pLKO.1-shRNAs in SNB19 cells. (**C**) Transwell assays were conducted to assess the effect of HOXA-AS3 knockdown on cell migration in SNB19 cells. Data were shown as mean ± SD.

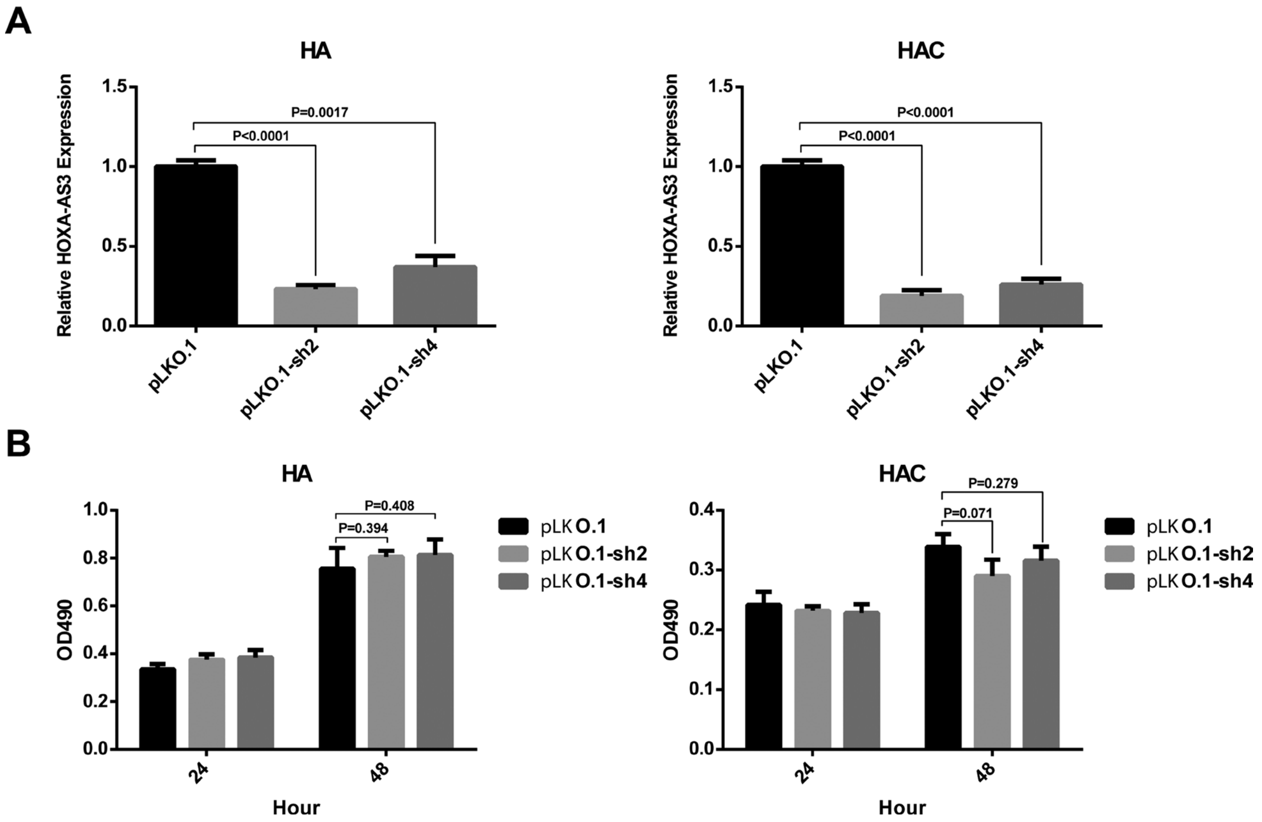

Supplementary Figure 3: Knockdown of HOXA-AS3 does not inhibit the growth of normal glial cells *in vitro*. (**A**) qPCR analysis of the knockdown efficiency of sh2 and sh4 in HA and HAC cells. (**B**) MTS assay was performed to determine the cell viability after transfenction with pLKO.1 or pLKO.1-shRNAs. Data were shown as mean ± SD.

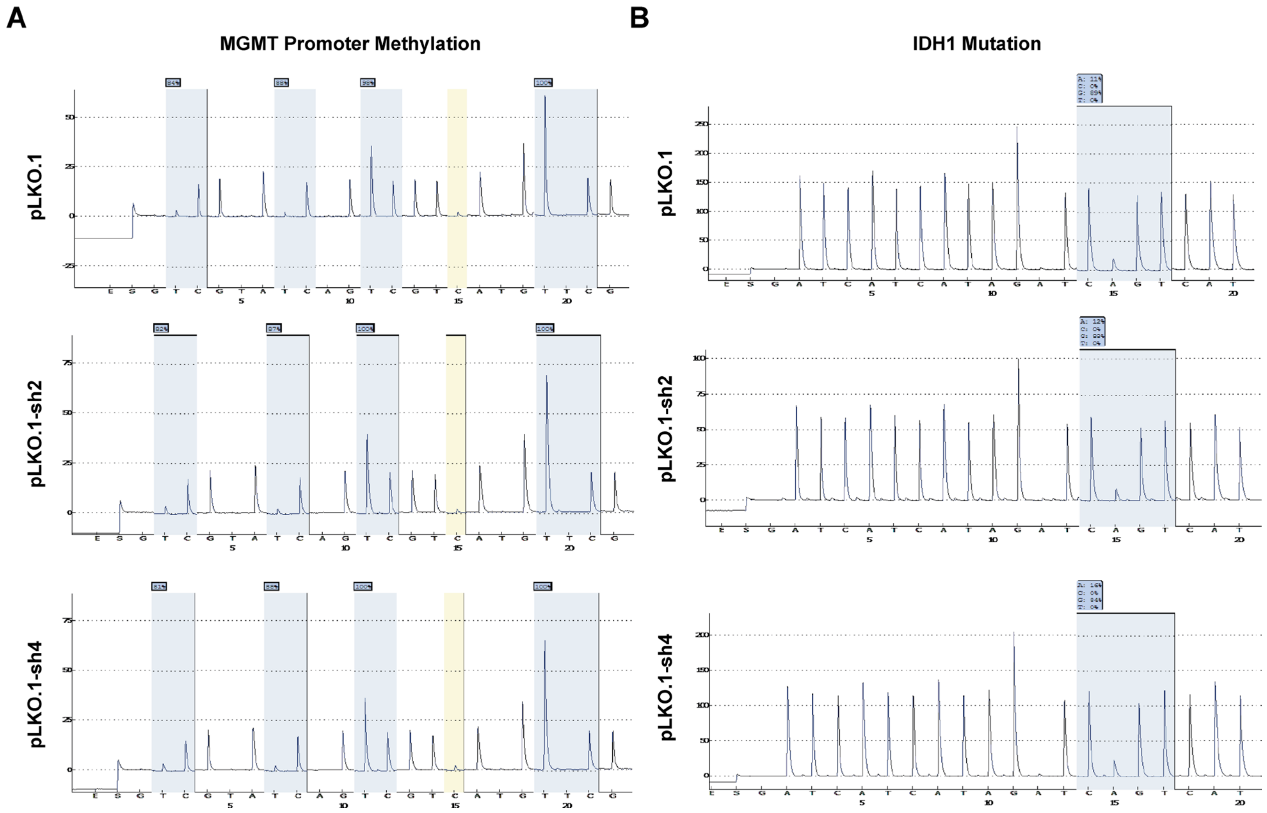

Supplementary Figure 4: Knockdown of HOXA-AS3 does not affect the IDH1 mutation and MGMT promoter methylation. (**A**) Pyrosequencing was performed to detect the methylation of MGMT promoter with stable LN229 cell lines (pLKO.1, pLKO.1-sh2 and pLKO.1-sh4). (**B**) DNA sequencing was used to exam the IDH1 mutation (R132H).

Supplementary Table 1: qPCR primer sequences

**HOXA-AS3**
Forward: 5′-GCTGAATTAACGGTGGCTCC-3′
Reverse: 5′-ATGGCGAGCGAAGGG AAG-3′
GAPDH
Forward: 5′- TTAAAAGCAGCCCTGGTGAC-3′
Reverse: 5′-CTCTGCTCCTCCTGTTCGAC-3′
HOXA-AS3 shRNAs sequences
HOXA-AS3-sh2
Sense: 5′-CCGGCTCTGGCTGAATTAACGGTCTCGAGACCGTTAATTCAGCCA GAGTTTTTG-3′
Anti-sense: 5′-AATTCAAAAACTCTGGCTGAATTAACGGTCTCGAGACCGTTA ATTCAGCCAGAG-3′
HOXA-AS3-sh4
Sense: 5′- CCGGGGATCTAGAAGATGTTAGTCTCGAGACTAACATCTTCTAGA TCCTTTTTG-3′
Anti-sense: 5′- AATTCAAAAAGGATCTAGAAGATGTTAGTCTCGAGACTAACA TCTTCTAGATCC-3′

Supplementary Table 2: The list of 500 genes positively and negatively associated with HOXA-AS3.
See Supplementary_Table_2
