# Supplementary material for: Upregulation of long noncoding RNA HOXA-AS3 promotes tumor progression and predicts poor prognosis in glioma
Source: Oncotarget. 2017 May 24;8(32):53110–23. doi: 10.18632/oncotarget.18162 (PMC5581096; doi:10.18632/oncotarget.18162)
Supplement: Supplementary file 2 [file oncotarget-08-53110-s002.docx]

**S1.2: The list of 500 genes positively and negatively associated with HOXA-AS3.**

| Gene Name | Fold Change | q-value(%) |  |
| --- | --- | --- | --- |
| MYL1 | 56.679 | 4.899 | positive |
| HOXA3 | 53.455 | 0 |  |
| HOXA5 | 39.228 | 0 |  |
| HOXA4 | 33.842 | 0 |  |
| OTOR | 32.33 | 3.394 |  |
| HOXC-AS5 | 30.05 | 0 |  |
| PISRT1 | 28.503 | 1.379 |  |
| EPYC | 28.314 | 3.394 |  |
| CAPN6 | 25.615 | 2.254 |  |
| MIR205 | 22.668 | 0.072 |  |
| MMP13 | 21.74 | 2.254 |  |
| C6orf15 | 20.191 | 0.359 |  |
| PITX2 | 16.749 | 0.072 |  |
| HOXA9 | 16.494 | 0 |  |
| HOXA7 | 16.462 | 0 |  |
| HOXA-AS4 | 16.277 | 0 |  |
| MIR192 | 16.232 | 0.022 |  |
| HOXA6 | 15.41 | 0 |  |
| REG1A | 15.045 | 3.394 |  |
| UNCX | 14.956 | 2.254 |  |
| LOC100506013 | 13.771 | 0.022 |  |
| HOXA2 | 13.27 | 0 |  |
| TNNI1 | 13.183 | 3.394 |  |
| GATA4 | 12.493 | 0 |  |
| C11orf88 | 12.281 | 0.359 |  |
| TMPRSS2 | 12.246 | 3.394 |  |
| HOTAIR | 12.161 | 0 |  |
| LGALS7 | 12.064 | 0.179 |  |
| HOXC13 | 11.861 | 0 |  |
| MS4A13 | 11.707 | 0.359 |  |
| KIR2DL2 | 11.543 | 0.734 |  |
| TRIM48 | 11.383 | 0.734 |  |
| MIR183 | 11.274 | 2.254 |  |
| HOXC10 | 11.27 | 0 |  |
| IL19 | 10.26 | 0.072 |  |
| LINC00608 | 10.21 | 3.394 |  |
| HOXD10 | 10.159 | 0 |  |
| MIR5194 | 10.149 | 2.254 |  |
| TMPRSS11A | 9.996 | 0.359 |  |
| POU6F2-AS1 | 9.954 | 2.254 |  |
| HOXA10 | 9.914 | 0 |  |
| FAM9A | 9.776 | 4.899 |  |
| HOXD11 | 9.621 | 0 |  |
| SCGB2A1 | 9.539 | 4.899 |  |
| LOC146513 | 9.512 | 2.254 |  |
| MIR1289-2 | 9.414 | 2.254 |  |
| HOXA10-HOXA9 | 8.838 | 0.179 |  |
| PI3 | 8.818 | 0.179 |  |
| FLJ25758 | 8.696 | 4.899 |  |
| HP | 8.634 | 0.072 |  |
| BPIFA3 | 8.495 | 2.254 |  |
| NKX2-5 | 8.409 | 0 |  |
| HOTAIRM1 | 8.39 | 0 |  |
| KERA | 8.365 | 1.379 |  |
| SLC44A4 | 8.335 | 2.254 |  |
| SCARNA14 | 8.286 | 0.359 |  |
| LOC100288255 | 8.279 | 4.899 |  |
| BPIFB4 | 8.145 | 0.734 |  |
| HOXD13 | 8.14 | 0 |  |
| DEFA8P | 8.023 | 2.254 |  |
| SAA2-SAA4 | 7.945 | 0.072 |  |
| LINC00559 | 7.867 | 0.734 |  |
| PRDM13 | 7.829 | 0 |  |
| IL22RA2 | 7.702 | 1.379 |  |
| CPXCR1 | 7.542 | 0.734 |  |
| MEOX2 | 7.454 | 0 |  |
| KRTAP17-1 | 7.454 | 5.556 |  |
| CCDC83 | 7.345 | 0.734 |  |
| IL4 | 7.304 | 0.072 |  |
| GABRR1 | 7.283 | 0.734 |  |
| OTP | 7.266 | 0 |  |
| GSG1 | 7.239 | 4.899 |  |
| HOXC11 | 6.945 | 0 |  |
| CCL20 | 6.917 | 0.734 |  |
| MEOX1 | 6.881 | 4.899 |  |
| MIR3944 | 6.787 | 1.379 |  |
| TPO | 6.656 | 3.394 |  |
| LGALS12 | 6.651 | 0 |  |
| HOXB3 | 6.636 | 0 |  |
| COL2A1 | 6.616 | 2.254 |  |
| UGT2B28 | 6.598 | 0.734 |  |
| LINC00475 | 6.549 | 0 |  |
| LCE2C | 6.54 | 0.022 |  |
| CBLC | 6.529 | 0.022 |  |
| LHX8 | 6.527 | 0.359 |  |
| KCTD14 | 6.466 | 0 |  |
| BANCR | 6.455 | 3.394 |  |
| HOXD9 | 6.38 | 0 |  |
| NRK | 6.377 | 0.734 |  |
| HOXB4 | 6.354 | 0 |  |
| HOXC9 | 6.353 | 0 |  |
| C20orf85 | 6.335 | 2.254 |  |
| SAA4 | 6.312 | 0.734 |  |
| PHOX2B | 6.254 | 0.734 |  |
| ORM1 | 6.252 | 1.379 |  |
| GPR1 | 6.252 | 1.379 |  |
| HOXA11 | 6.25 | 0 |  |
| FLJ41350 | 6.153 | 0 |  |
| TNMD | 6.139 | 4.899 |  |
| FEZF1-AS1 | 6.121 | 0 |  |
| CALCB | 6.107 | 4.899 |  |
| AA06 | 6.087 | 4.899 |  |
| HOXA11-AS | 6.081 | 0 |  |
| NNMT | 6.071 | 0 |  |
| LINC00550 | 6.026 | 2.254 |  |
| REG1B | 6.008 | 0.072 |  |
| HOXA1 | 5.986 | 0 |  |
| SAA2 | 5.972 | 0.359 |  |
| LILRA3 | 5.969 | 0.359 |  |
| EREG | 5.932 | 1.379 |  |
| COMP | 5.932 | 3.394 |  |
| CA3 | 5.928 | 0.072 |  |
| CCL7 | 5.915 | 1.379 |  |
| TTR | 5.898 | 4.899 |  |
| KCNMB2-IT1 | 5.851 | 0.359 |  |
| MYOG | 5.785 | 4.899 |  |
| HOXB13 | 5.753 | 0.005 |  |
| WT1-AS | 5.741 | 0 |  |
| LINC00841 | 5.687 | 2.254 |  |
| LOC100506229 | 5.682 | 1.379 |  |
| C12orf56 | 5.666 | 4.899 |  |
| KCNJ15 | 5.653 | 0.359 |  |
| TECRL | 5.63 | 4.899 |  |
| LOC145845 | 5.599 | 0.359 |  |
| SHOX | 5.559 | 5.556 |  |
| HOXC12 | 5.548 | 0.179 |  |
| FEZF1 | 5.525 | 1.379 |  |
| DMBX1 | 5.497 | 0 |  |
| TCF21 | 5.485 | 3.394 |  |
| CCL11 | 5.472 | 1.379 |  |
| POU4F1 | 5.463 | 0.734 |  |
| CCDC19 | 5.423 | 0.005 |  |
| MIR506 | 5.4 | 0.734 |  |
| RPS7P5 | 5.365 | 3.394 |  |
| LYZL6 | 5.358 | 0.359 |  |
| FLJ22763 | 5.353 | 2.254 |  |
| LGALS16 | 5.352 | 0.734 |  |
| OSR2 | 5.35 | 0 |  |
| KRT75 | 5.33 | 0 |  |
| ASZ1 | 5.33 | 4.899 |  |
| TMEM8C | 5.32 | 3.394 |  |
| SEC61G | 5.291 | 0 |  |
| MIR3976 | 5.282 | 0.072 |  |
| BPIFC | 5.264 | 4.899 |  |
| SHOX2 | 5.246 | 0 |  |
| C12orf77 | 5.232 | 3.394 |  |
| MSMB | 5.231 | 0.734 |  |
| WFDC9 | 5.23 | 0.072 |  |
| MYLPF | 5.217 | 2.254 |  |
| RCAN3AS | 5.197 | 2.254 |  |
| DNM3OS | 5.168 | 1.379 |  |
| LOC100131635 | 5.158 | 2.254 |  |
| CCL13 | 5.156 | 0.005 |  |
| CST2 | 5.131 | 3.394 |  |
| OS9 | 5.098 | 0.072 |  |
| GP2 | 5.088 | 4.899 |  |
| MIR34A | 5.045 | 0.072 |  |
| CYP2C8 | 5.036 | 1.379 |  |
| LGALS13 | 5.012 | 0.734 |  |
| FAM74A1 | 4.965 | 0.734 |  |
| UGT2B17 | 4.955 | 0.359 |  |
| IGFBP2 | 4.944 | 0 |  |
| OR2A4 | 4.915 | 4.899 |  |
| KRTAP9-8 | 4.889 | 1.379 |  |
| OR8G1 | 4.886 | 0.072 |  |
| MIR1284 | 4.873 | 4.899 |  |
| SNORA11C | 4.864 | 4.899 |  |
| TPRG1-AS2 | 4.863 | 0.022 |  |
| STOML3 | 4.863 | 0.734 |  |
| GPR15 | 4.859 | 3.394 |  |
| HGD | 4.856 | 0 |  |
| VGLL2 | 4.854 | 0 |  |
| PRRX2 | 4.851 | 0 |  |
| HOXC6 | 4.848 | 0 |  |
| IL8 | 4.839 | 0.734 |  |
| LOC1720 | 4.832 | 0.179 |  |
| SLC23A1 | 4.83 | 2.254 |  |
| TDO2 | 4.827 | 0.005 |  |
| TMCO5A | 4.825 | 0.005 |  |
| HDC | 4.816 | 0 |  |
| FAM99B | 4.816 | 4.899 |  |
| CELA3A | 4.814 | 2.254 |  |
| TUBA3C | 4.787 | 1.379 |  |
| FCGR2B | 4.785 | 0.359 |  |
| KRTAP21-3 | 4.784 | 0.022 |  |
| KRTAP20-1 | 4.775 | 0.179 |  |
| SIM1 | 4.77 | 0.022 |  |
| EPO | 4.769 | 1.379 |  |
| SAA1 | 4.725 | 0.022 |  |
| KRT13 | 4.672 | 1.379 |  |
| LINC00351 | 4.668 | 3.394 |  |
| FRG2B | 4.666 | 3.394 |  |
| NBLA00301 | 4.639 | 0 |  |
| HOXB7 | 4.619 | 0 |  |
| HOXB8 | 4.594 | 0.179 |  |
| MIR3907 | 4.589 | 0 |  |
| EN1 | 4.586 | 0 |  |
| PITX1 | 4.569 | 0 |  |
| CYP2C9 | 4.567 | 2.254 |  |
| C19orf59 | 4.56 | 0.359 |  |
| HIST1H2AJ | 4.548 | 0.005 |  |
| FGG | 4.546 | 0.005 |  |
| DCD | 4.538 | 2.254 |  |
| AGR3 | 4.519 | 0.359 |  |
| TEKT4 | 4.507 | 3.394 |  |
| STAC | 4.49 | 0 |  |
| PRSS37 | 4.487 | 0.734 |  |
| MMP1 | 4.484 | 0.179 |  |
| LINC00333 | 4.482 | 2.254 |  |
| HAND2 | 4.481 | 0 |  |
| AGXT | 4.481 | 0 |  |
| COL6A5 | 4.479 | 5.556 |  |
| LIMS3-LOC440895 | 4.468 | 0.734 |  |
| SP8 | 4.466 | 0 |  |
| LINC00254 | 4.464 | 4.899 |  |
| LINC00307 | 4.456 | 3.394 |  |
| C15orf43 | 4.448 | 0 |  |
| LOC100499194 | 4.444 | 0.022 |  |
| PRSS58 | 4.432 | 0 |  |
| PRL | 4.426 | 0.179 |  |
| CHODL | 4.418 | 0.179 |  |
| CAPSL | 4.405 | 0.359 |  |
| CA6 | 4.401 | 2.254 |  |
| UGT2A3 | 4.377 | 2.254 |  |
| CDHR4 | 4.376 | 0.359 |  |
| LOC100130776 | 4.367 | 0.179 |  |
| DMRTA2 | 4.363 | 0 |  |
| OR52E6 | 4.362 | 0 |  |
| SULT1E1 | 4.362 | 0.734 |  |
| COL1A1 | 4.36 | 0.022 |  |
| MAT1A | 4.338 | 4.899 |  |
| CSRP3 | 4.325 | 3.394 |  |
| TFF3 | 4.32 | 0.734 |  |
| GSTA1 | 4.316 | 1.379 |  |
| EGFL6 | 4.315 | 0.734 |  |
| IL1F10 | 4.314 | 0.022 |  |
| MYH8 | 4.311 | 0.359 |  |
| LUM | 4.309 | 0.179 |  |
| LINC00474 | 4.3 | 3.394 |  |
| OR6C75 | 4.297 | 0.179 |  |
| FAM74A4 | 4.275 | 0.734 |  |
| ACMSD | 4.268 | 5.556 |  |
| LGR6 | 4.255 | 0.359 |  |
| C6 | 4.25 | 3.394 |  |
| IGF2BP3 | 4.245 | 0 |  |
| POSTN | 4.23 | 0.022 |  |
| FAM74A3 | 4.23 | 0.734 |  |
| SNORA30 | 4.215 | 0.734 |  |
| G0S2 | 4.212 | 0 |  |
| SLAMF9 | 4.212 | 0 |  |
| WISP1 | 4.206 | 0 |  |
| FTLP10 | 4.187 | 0.179 |  |
| HIST1H3I | 4.186 | 0 |  |
| CA9 | 4.185 | 0 |  |
| LOC644649 | 4.185 | 0.072 |  |
| LYZL2 | 4.185 | 2.254 |  |
| CXCL5 | 4.171 | 1.379 |  |
| CCDC140 | 4.163 | 0 |  |
| ADAM3A | 4.157 | 4.899 |  |
| LBX1 | 4.153 | 0 |  |
| NKX3-2 | 4.135 | 0 |  |
| SPACA1 | 4.134 | 0 |  |
| RETN | 4.134 | 0.005 |  |
| OR5R1 | 4.118 | 0.005 |  |
| SPATA31D4 | 4.116 | 0.179 |  |
| PCOLCE | 4.109 | 0 |  |
| MIR663B | 4.109 | 0.359 |  |
| CHRND | 4.108 | 0.072 |  |
| LOC100129175 | 4.097 | 0.072 |  |
| TTC26 | 4.096 | 0.022 |  |
| IGF2BP1 | 4.088 | 0.022 |  |
| HOXB2 | 4.081 | 0 |  |
| C7orf69 | 4.079 | 0.359 |  |
| COL1A2 | 4.075 | 0.005 |  |
| C8orf22 | 4.072 | 0.179 |  |
| IL36G | 4.052 | 0.005 |  |
| C15orf48 | 4.036 | 0.072 |  |
| UGT2B7 | 4.031 | 0.005 |  |
| IBSP | 4.027 | 0 |  |
| EIF3IP1 | 4.004 | 0.072 |  |
| CD70 | 3.991 | 0.734 |  |
| MYBPH | 3.988 | 0.072 |  |
| CTSL3P | 3.985 | 0 |  |
| LOC340094 | 3.985 | 1.379 |  |
| CKM | 3.983 | 3.394 |  |
| EDN2 | 3.979 | 0.022 |  |
| MYL4 | 3.973 | 0.179 |  |
| MAEL | 3.971 | 4.899 |  |
| CLDN14 | 3.953 | 0.005 |  |
| COL6A2 | 3.946 | 0 |  |
| CHODL-AS1 | 3.936 | 1.379 |  |
| LINC00353 | 3.932 | 4.899 |  |
| TUBA4B | 3.923 | 0.072 |  |
| HOXB5 | 3.918 | 0.005 |  |
| LINC00651 | 3.914 | 0.359 |  |
| ORC1 | 3.907 | 0 |  |
| PPBP | 3.903 | 0.072 |  |
| LINC00669 | 3.902 | 0 |  |
| ZP2 | 3.901 | 0.072 |  |
| HTN1 | 3.901 | 0.179 |  |
| SIX6 | 3.897 | 0 |  |
| DPPA2 | 3.897 | 0.359 |  |
| LOC285547 | 3.891 | 0.734 |  |
| ABCC3 | 3.889 | 0 |  |
| CRABP1 | 3.883 | 0.179 |  |
| IL36B | 3.878 | 0.179 |  |
| LINC00668 | 3.874 | 0.005 |  |
| MYL10 | 3.87 | 0.359 |  |
| HIST1H2BI | 3.869 | 0 |  |
| COLQ | 3.864 | 0 |  |
| MMP9 | 3.863 | 0.072 |  |
| OR8B3 | 3.85 | 2.254 |  |
| GPR139 | 3.838 | 0 |  |
| CYLC2 | 3.836 | 0 |  |
| ZNF683 | 3.836 | 1.379 |  |
| PDX1 | 3.832 | 1.379 |  |
| H19 | 3.825 | 0.359 |  |
| TAAR1 | 3.824 | 0.005 |  |
| HIST1H2BH | 3.821 | 0 |  |
| BARHL1 | 3.819 | 5.556 |  |
| SDC1 | 3.818 | 0.022 |  |
| SEMG2 | 3.808 | 0.022 |  |
| PIH1D3 | 3.797 | 1.379 |  |
| CPA6 | 3.792 | 0.734 |  |
| GC | 3.791 | 0.734 |  |
| POU1F1 | 3.789 | 0.179 |  |
| NXPH4 | 3.78 | 0 |  |
| TOP1P2 | 3.773 | 2.254 |  |
| CCKAR | 3.772 | 0 |  |
| CD300E | 3.772 | 0 |  |
| GH1 | 3.768 | 0.022 |  |
| C1orf189 | 3.76 | 0.359 |  |
| LINC00052 | 3.76 | 4.899 |  |
| OR51S1 | 3.758 | 0.072 |  |
| MAGEA6 | 3.757 | 2.254 |  |
| OR4A16 | 3.755 | 0.734 |  |
| TEAD2 | 3.754 | 0 |  |
| LOC440117 | 3.749 | 1.379 |  |
| ISL2 | 3.742 | 0 |  |
| CDX2 | 3.734 | 0.179 |  |
| HSD3B1 | 3.73 | 0.179 |  |
| TRPM1 | 3.726 | 0.022 |  |
| DEFB135 | 3.725 | 0.005 |  |
| BPIFB3 | 3.721 | 0.005 |  |
| LOC340357 | 3.72 | 3.394 |  |
| OR6C6 | 3.718 | 0.005 |  |
| TIMP1 | 3.711 | 0 |  |
| NF1P2 | 3.709 | 0.005 |  |
| UGT2A2 | 3.709 | 0.022 |  |
| LINC00160 | 3.704 | 0.022 |  |
| HIST1H2BM | 3.695 | 0 |  |
| SPINK14 | 3.692 | 0.359 |  |
| LEP | 3.688 | 0 |  |
| HIST1H3F | 3.688 | 0.072 |  |
| TWIST2 | 3.682 | 0.005 |  |
| EYA4 | 3.681 | 0 |  |
| LOC285548 | 3.681 | 0.005 |  |
| LGALS8 | 3.673 | 3.394 |  |
| DIRC1 | 3.672 | 0 |  |
| GLYCAM1 | 3.66 | 0.022 |  |
| LTF | 3.658 | 0.359 |  |
| AKAP4 | 3.657 | 0.734 |  |
| TM4SF19 | 3.656 | 0.022 |  |
| C7orf76 | 3.653 | 5.556 |  |
| OR52L1 | 3.648 | 0.022 |  |
| CSF2 | 3.648 | 0.359 |  |
| FAM19A3 | 3.647 | 0.005 |  |
| GSTA5 | 3.645 | 0.005 |  |
| DEFB128 | 3.645 | 0.179 |  |
| OR51V1 | 3.638 | 0.022 |  |
| ZCCHC13 | 3.637 | 0.359 |  |
| BSX | 3.632 | 0.072 |  |
| ADM | 3.627 | 0 |  |
| ESPNL | 3.622 | 0 |  |
| OR8D1 | 3.618 | 0.072 |  |
| FMO9P | 3.613 | 0 |  |
| PPP1R14D | 3.613 | 3.394 |  |
| CA1 | 3.61 | 1.379 |  |
| POTEC | 3.607 | 3.394 |  |
| COL3A1 | 3.606 | 0.022 |  |
| ARL14EPL | 3.604 | 0 |  |
| KRTAP4-6 | 3.604 | 0.734 |  |
| OR1S2 | 3.602 | 0.005 |  |
| OR5I1 | 3.602 | 0.005 |  |
| LOX | 3.601 | 0 |  |
| IGFBP3 | 3.601 | 0 |  |
| OR6C76 | 3.598 | 0.179 |  |
| KRT8 | 3.597 | 0.022 |  |
| HEMGN | 3.594 | 0.734 |  |
| UGT2B10 | 3.592 | 0.022 |  |
| PDC | 3.591 | 0.359 |  |
| ZNF180 | 3.589 | 4.899 |  |
| TMEM207 | 3.587 | 0 |  |
| OR8B12 | 3.579 | 0 |  |
| ADM2 | 3.575 | 0 |  |
| OR2T11 | 3.563 | 0.005 |  |
| TCF23 | 3.562 | 0 |  |
| NOS2 | 3.562 | 0.734 |  |
| HIST1H3G | 3.56 | 0 |  |
| ATP4B | 3.553 | 1.379 |  |
| IKZF1 | 3.546 | 1.379 |  |
| EDDM3A | 3.545 | 0 |  |
| BMP5 | 3.545 | 2.254 |  |
| KRTAP4-8 | 3.544 | 0.022 |  |
| PTX3 | 3.52 | 0.072 |  |
| ROPN1L | 3.516 | 0.359 |  |
| VN1R10P | 3.515 | 0 |  |
| CT64 | 3.515 | 4.899 |  |
| EN2 | 3.513 | 0 |  |
| HIST1H3C | 3.513 | 0 |  |
| GALNTL5 | 3.513 | 0.022 |  |
| OR6C65 | 3.507 | 0.179 |  |
| OR5M3 | 3.505 | 0.005 |  |
| ARHGEF3-AS1 | 3.494 | 1.379 |  |
| MYBL2 | 3.488 | 0 |  |
| GALP | 3.486 | 0.359 |  |
| HOXC4 | 3.484 | 0 |  |
| JRKL-AS1 | 3.484 | 4.899 |  |
| OR8K3 | 3.482 | 0.005 |  |
| M1 | 3.481 | 3.394 |  |
| MMP8 | 3.474 | 0.734 |  |
| SPO11 | 3.473 | 0.734 |  |
| LOC728012 | 3.473 | 4.899 |  |
| HMX3 | 3.468 | 0.179 |  |
| EMILIN3 | 3.462 | 0 |  |
| MIR548K | 3.461 | 3.394 |  |
| POTEA | 3.461 | 3.394 |  |
| MIR941-1 | 3.46 | 2.254 |  |
| OR9A4 | 3.457 | 0.072 |  |
| AKR1CL1 | 3.456 | 1.379 |  |
| OR4K13 | 3.454 | 0 |  |
| MT1B | 3.453 | 0 |  |
| GALNT5 | 3.452 | 0 |  |
| TOPAZ1 | 3.451 | 0.005 |  |
| EGFR | 3.45 | 0 |  |
| RNF223 | 3.449 | 0 |  |
| LYPD4 | 3.445 | 0.179 |  |
| COL5A1 | 3.443 | 0 |  |
| WFDC12 | 3.443 | 0 |  |
| OR5K3 | 3.442 | 0.005 |  |
| CXCL3 | 3.442 | 0.359 |  |
| LOC375295 | 3.436 | 0 |  |
| OR2A14 | 3.433 | 0.005 |  |
| FMO3 | 3.429 | 0.734 |  |
| TPSB2 | 3.425 | 0.179 |  |
| LOC100506474 | 3.422 | 0 |  |
| SLC22A7 | 3.421 | 1.379 |  |
| TREML3P | 3.414 | 0 |  |
| XG | 3.41 | 1.379 |  |
| C4BPA | 3.409 | 3.394 |  |
| MMP19 | 3.408 | 0 |  |
| OR8B4 | 3.402 | 0.005 |  |
| KRTAP20-4 | 3.401 | 0.022 |  |
| F2RL2 | 3.401 | 0.022 |  |
| SCNN1G | 3.397 | 0 |  |
| RAET1K | 3.396 | 0 |  |
| TAS2R38 | 3.396 | 0 |  |
| C3orf30 | 3.394 | 0.005 |  |
| C9orf117 | 3.393 | 1.379 |  |
| DBX1 | 3.39 | 3.394 |  |
| OR8J1 | 3.387 | 0.005 |  |
| LOC643441 | 3.387 | 0.359 |  |
| FGF23 | 3.387 | 3.394 |  |
| LINC00583 | 3.385 | 0.072 |  |
| BIRC8 | 3.384 | 0 |  |
| OR4B1 | 3.379 | 0.005 |  |
| LOC649330 | 3.377 | 0.022 |  |
| OR6N2 | 3.376 | 0.734 |  |
| HTR3E | 3.373 | 0.072 |  |
| STEAP3 | 3.369 | 0 |  |
| OR1A2 | 3.368 | 0.022 |  |
| TSHB | 3.368 | 0.072 |  |
| OR2W1 | 3.366 | 0.005 |  |
| HSPA6 | 3.362 | 0.022 |  |
| HIST1H1D | 3.361 | 0 |  |
| LOC283914 | 3.359 | 0.359 |  |
| KRTAP5-3 | 3.356 | 0.734 |  |
| CLEC5A | 3.355 | 0 |  |
| HIST1H2AL | 3.354 | 0 |  |
| CT47B1 | 3.354 | 0.005 |  |
| HIST1H2BA | 3.352 | 0.005 |  |
| OR52A1 | 3.349 | 0.022 |  |
| DEFA4 | 3.345 | 0 |  |
| DEFB110 | 3.344 | 0.005 |  |
| SERPINB11 | 3.344 | 0.179 |  |
| COL6A3 | 3.343 | 0.072 |  |
| UGT1A7 | 3.342 | 0.005 |  |
| KISS1R | 3.341 | 0 |  |
| HEPACAM2 | 3.341 | 3.394 |  |
| DPPA2P3 | 3.34 | 0 |  |
| C5orf46 | 3.34 | 0.072 |  |
| UGT2B11 | 3.339 | 0.022 |  |
| SNTN | 3.336 | 0.179 |  |
| TULP1 | 3.335 | 0 |  |
| BHLHE23 | 3.332 | 0.022 |  |
| ASB15 | 3.331 | 0 |  |
| MBL2 | 3.329 | 0 |  |
| UBE2U | 3.325 | 0 |  |
| OR2D3 | 3.325 | 0.005 |  |
| ITIH6 | 3.324 | 0.072 |  |
| GLYR1 | 0.952 | 5.556 | negative |
| STARD7 | 0.947 | 5.556 |  |
| UBR2 | 0.944 | 3.394 |  |
| GID4 | 0.943 | 4.899 |  |
| ZMAT2 | 0.941 | 4.899 |  |
| THUMPD1 | 0.939 | 3.394 |  |
| EXOC8 | 0.939 | 5.556 |  |
| FAM219B | 0.938 | 3.394 |  |
| HECA | 0.936 | 4.899 |  |
| PIGH | 0.936 | 5.556 |  |
| STX12 | 0.934 | 3.394 |  |
| SAMM50 | 0.934 | 5.556 |  |
| MRPL46 | 0.934 | 5.556 |  |
| C16orf70 | 0.934 | 5.556 |  |
| SNX27 | 0.933 | 3.394 |  |
| EPG5 | 0.933 | 3.394 |  |
| TRAPPC4 | 0.933 | 4.899 |  |
| LOC100859930 | 0.932 | 4.899 |  |
| HEATR5B | 0.932 | 4.899 |  |
| FBXO8 | 0.932 | 4.899 |  |
| NUP133 | 0.932 | 5.556 |  |
| RBM41 | 0.931 | 3.394 |  |
| C22orf28 | 0.931 | 3.394 |  |
| THAP11 | 0.931 | 3.394 |  |
| MMGT1 | 0.93 | 2.254 |  |
| ZNF287 | 0.93 | 3.394 |  |
| SAT2 | 0.93 | 5.556 |  |
| TOX4 | 0.929 | 0.734 |  |
| VPS33B | 0.929 | 2.254 |  |
| COG1 | 0.929 | 3.394 |  |
| SLC25A46 | 0.929 | 3.394 |  |
| GRHPR | 0.929 | 4.899 |  |
| TMEM128 | 0.929 | 5.556 |  |
| PSMC6 | 0.929 | 5.556 |  |
| COA3 | 0.928 | 5.556 |  |
| HSD17B1 | 0.927 | 4.899 |  |
| C9orf85 | 0.927 | 4.899 |  |
| POLR3GL | 0.926 | 4.899 |  |
| FBXO45 | 0.926 | 5.556 |  |
| CIAO1 | 0.925 | 0 |  |
| KCTD2 | 0.925 | 2.254 |  |
| FEM1B | 0.925 | 3.394 |  |
| POLI | 0.925 | 4.899 |  |
| ARV1 | 0.925 | 4.899 |  |
| KIDINS220 | 0.925 | 4.899 |  |
| SPOPL | 0.925 | 4.899 |  |
| KIAA1328 | 0.925 | 5.556 |  |
| VPS11 | 0.924 | 0.359 |  |
| VPS39 | 0.924 | 0.359 |  |
| HADHA | 0.924 | 0.734 |  |
| GFM1 | 0.924 | 1.379 |  |
| CUL5 | 0.924 | 2.254 |  |
| CLUAP1 | 0.924 | 3.394 |  |
| NUCKS1 | 0.923 | 0.359 |  |
| TIMM10B | 0.923 | 1.379 |  |
| SLK | 0.923 | 3.394 |  |
| MRPS31 | 0.923 | 4.899 |  |
| DDX51 | 0.923 | 5.556 |  |
| ATP6V0D1 | 0.922 | 3.394 |  |
| ZSCAN29 | 0.922 | 4.899 |  |
| C14orf101 | 0.922 | 5.556 |  |
| SIK2 | 0.922 | 5.556 |  |
| FAM175B | 0.921 | 1.379 |  |
| NRDE2 | 0.921 | 3.394 |  |
| MPI | 0.92 | 0.734 |  |
| GLTSCR1L | 0.92 | 1.379 |  |
| FUBP3 | 0.92 | 1.379 |  |
| PTPMT1 | 0.92 | 4.899 |  |
| TMEM41B | 0.92 | 4.899 |  |
| EIF3F | 0.92 | 4.899 |  |
| NUDT21 | 0.92 | 5.556 |  |
| ATMIN | 0.919 | 0.359 |  |
| ZFR | 0.919 | 0.359 |  |
| DIMT1 | 0.919 | 1.379 |  |
| EIF3A | 0.919 | 3.394 |  |
| NDST1 | 0.919 | 3.394 |  |
| LACE1 | 0.919 | 5.556 |  |
| VPS4B | 0.918 | 0.359 |  |
| PPP1R2 | 0.918 | 1.379 |  |
| CCDC53 | 0.918 | 3.394 |  |
| XPNPEP3 | 0.918 | 3.394 |  |
| XPO4 | 0.918 | 3.394 |  |
| RAB11FIP3 | 0.918 | 4.899 |  |
| TIMM17B | 0.918 | 4.899 |  |
| RHOT1 | 0.918 | 5.556 |  |
| TCF25 | 0.917 | 0.359 |  |
| TERF2 | 0.917 | 0.734 |  |
| WAPAL | 0.917 | 2.254 |  |
| ISCA2 | 0.917 | 3.394 |  |
| TTLL4 | 0.917 | 3.394 |  |
| ERLIN2 | 0.916 | 2.254 |  |
| RABGAP1L | 0.916 | 4.899 |  |
| PET117 | 0.916 | 4.899 |  |
| YLPM1 | 0.916 | 5.556 |  |
| DECR1 | 0.915 | 1.379 |  |
| ABCA11P | 0.915 | 5.556 |  |
| SMG6 | 0.915 | 5.556 |  |
| LAMTOR1 | 0.914 | 0.359 |  |
| KPNA3 | 0.914 | 0.359 |  |
| AHSA1 | 0.914 | 0.734 |  |
| WIPF2 | 0.913 | 0.734 |  |
| PRDX3 | 0.913 | 3.394 |  |
| DNAJC15 | 0.913 | 3.394 |  |
| FGD5-AS1 | 0.913 | 5.556 |  |
| CCDC25 | 0.912 | 0.072 |  |
| PCYOX1 | 0.912 | 1.379 |  |
| EFCAB14 | 0.912 | 2.254 |  |
| PRDX5 | 0.912 | 3.394 |  |
| EP300 | 0.912 | 4.899 |  |
| MPHOSPH8 | 0.912 | 5.556 |  |
| CNOT8 | 0.911 | 0.359 |  |
| FAM8A1 | 0.911 | 0.359 |  |
| CCDC115 | 0.911 | 0.359 |  |
| WDR5B | 0.911 | 2.254 |  |
| SPIN1 | 0.911 | 2.254 |  |
| UBR4 | 0.911 | 3.394 |  |
| TADA2B | 0.91 | 0.072 |  |
| MRPL19 | 0.91 | 0.072 |  |
| TANGO6 | 0.91 | 0.734 |  |
| ATG14 | 0.91 | 1.379 |  |
| MFSD8 | 0.91 | 3.394 |  |
| SUV420H1 | 0.91 | 3.394 |  |
| PACSIN2 | 0.91 | 4.899 |  |
| ZNF688 | 0.91 | 5.556 |  |
| KDSR | 0.909 | 0.359 |  |
| WDR59 | 0.909 | 0.359 |  |
| RMND5B | 0.909 | 0.734 |  |
| RNF139 | 0.909 | 2.254 |  |
| BDP1 | 0.909 | 2.254 |  |
| VPS53 | 0.909 | 2.254 |  |
| ZFC3H1 | 0.909 | 3.394 |  |
| SETD7 | 0.909 | 3.394 |  |
| ZNF91 | 0.909 | 5.556 |  |
| SUCLG1 | 0.908 | 0.179 |  |
| BLOC1S6 | 0.908 | 0.734 |  |
| FLJ33630 | 0.908 | 4.899 |  |
| FRS3 | 0.908 | 5.556 |  |
| HIF1AN | 0.907 | 0.072 |  |
| MRPL30 | 0.907 | 0.179 |  |
| PSMD10 | 0.907 | 0.179 |  |
| ANAPC13 | 0.907 | 1.379 |  |
| RNF187 | 0.907 | 1.379 |  |
| APPL1 | 0.907 | 1.379 |  |
| ARFGEF2 | 0.907 | 1.379 |  |
| MGAT5 | 0.907 | 2.254 |  |
| TSPAN5 | 0.907 | 3.394 |  |
| KLHL24 | 0.907 | 3.394 |  |
| ASB8 | 0.906 | 0 |  |
| CDC40 | 0.906 | 1.379 |  |
| PARL | 0.906 | 1.379 |  |
| ZNF592 | 0.906 | 1.379 |  |
| NR2C2 | 0.906 | 1.379 |  |
| C5orf42 | 0.906 | 3.394 |  |
| YTHDC1 | 0.906 | 3.394 |  |
| LYPLAL1 | 0.906 | 3.394 |  |
| FLYWCH1 | 0.906 | 4.899 |  |
| KIN | 0.906 | 5.556 |  |
| FAM168B | 0.905 | 0.179 |  |
| GABARAP | 0.905 | 0.734 |  |
| SFXN1 | 0.905 | 1.379 |  |
| CRADD | 0.905 | 2.254 |  |
| AGPAT5 | 0.905 | 3.394 |  |
| ISCA1 | 0.905 | 4.899 |  |
| SS18L1 | 0.905 | 4.899 |  |
| LARS2 | 0.904 | 0.734 |  |
| ZNF710 | 0.904 | 0.734 |  |
| CCDC6 | 0.904 | 1.379 |  |
| CHD6 | 0.904 | 1.379 |  |
| PCNX | 0.904 | 1.379 |  |
| ITSN1 | 0.904 | 2.254 |  |
| DTWD2 | 0.904 | 2.254 |  |
| MBD5 | 0.904 | 3.394 |  |
| LOC286437 | 0.904 | 3.394 |  |
| RMDN1 | 0.904 | 3.394 |  |
| THAP9 | 0.904 | 4.899 |  |
| TET2 | 0.904 | 4.899 |  |
| BMS1P4 | 0.904 | 5.556 |  |
| PPM1B | 0.904 | 5.556 |  |
| SPPL3 | 0.903 | 0.179 |  |
| VCPIP1 | 0.903 | 0.359 |  |
| VTI1B | 0.903 | 0.359 |  |
| ANGEL2 | 0.903 | 0.359 |  |
| ECHS1 | 0.903 | 0.359 |  |
| SMCR7L | 0.903 | 0.734 |  |
| SEL1L | 0.903 | 0.734 |  |
| SPTBN1 | 0.903 | 1.379 |  |
| LOH12CR1 | 0.903 | 2.254 |  |
| NUFIP1 | 0.903 | 2.254 |  |
| COX7C | 0.903 | 4.899 |  |
| HNRNPUL2 | 0.902 | 0.359 |  |
| PRPF6 | 0.902 | 0.734 |  |
| TADA1 | 0.902 | 1.379 |  |
| WWC2 | 0.902 | 3.394 |  |
| TMEM256 | 0.902 | 3.394 |  |
| EXOC3 | 0.901 | 0.022 |  |
| UTP23 | 0.901 | 0.734 |  |
| UBQLN4 | 0.901 | 2.254 |  |
| EEF1E1 | 0.901 | 4.899 |  |
| CPSF2 | 0.9 | 0.072 |  |
| FAHD2A | 0.9 | 0.179 |  |
| CHUK | 0.9 | 0.734 |  |
| USP30 | 0.9 | 1.379 |  |
| FN3KRP | 0.9 | 1.379 |  |
| MTMR6 | 0.9 | 1.379 |  |
| VPS26A | 0.9 | 1.379 |  |
| SELK | 0.9 | 2.254 |  |
| NBN | 0.9 | 2.254 |  |
| PTGES2 | 0.9 | 3.394 |  |
| ALG11 | 0.9 | 4.899 |  |
| PIK3R4 | 0.899 | 0.005 |  |
| ACTR1B | 0.899 | 0.179 |  |
| DCUN1D1 | 0.899 | 0.359 |  |
| RAB28 | 0.899 | 0.734 |  |
| ACTR10 | 0.899 | 1.379 |  |
| ZER1 | 0.899 | 1.379 |  |
| EIF3H | 0.899 | 1.379 |  |
| RDH11 | 0.899 | 2.254 |  |
| CDK19 | 0.899 | 4.899 |  |
| GDF11 | 0.899 | 5.556 |  |
| WDR11 | 0.898 | 0.179 |  |
| DPY19L4 | 0.898 | 1.379 |  |
| UPF3A | 0.898 | 1.379 |  |
| ARID4A | 0.898 | 3.394 |  |
| ZNF425 | 0.898 | 3.394 |  |
| SPATA7 | 0.898 | 5.556 |  |
| NDUFA10 | 0.897 | 0.005 |  |
| KIAA1143 | 0.897 | 0.072 |  |
| EIF1AX | 0.897 | 0.734 |  |
| SIRT3 | 0.897 | 4.899 |  |
| EID1 | 0.896 | 0.072 |  |
| ZXDC | 0.896 | 0.179 |  |
| CNIH | 0.896 | 0.734 |  |
| DTD2 | 0.896 | 0.734 |  |
| BBS1 | 0.896 | 1.379 |  |
| C9orf123 | 0.896 | 1.379 |  |
| MRPL45 | 0.896 | 2.254 |  |
| ZNF624 | 0.896 | 2.254 |  |
| TATDN3 | 0.896 | 3.394 |  |
| PHAX | 0.895 | 0 |  |
| MAP1LC3B | 0.895 | 0.022 |  |
| UBR1 | 0.895 | 0.179 |  |
| COMMD9 | 0.895 | 0.359 |  |
| MLYCD | 0.895 | 0.359 |  |
| ABHD12 | 0.895 | 0.734 |  |
| LINC00667 | 0.895 | 2.254 |  |
| EEF1A1 | 0.895 | 2.254 |  |
| TMEM175 | 0.895 | 3.394 |  |
| RPL7 | 0.895 | 4.899 |  |
| C17orf59 | 0.894 | 0.072 |  |
| QRSL1 | 0.894 | 0.072 |  |
| WHSC1L1 | 0.894 | 0.359 |  |
| PYCR2 | 0.894 | 0.359 |  |
| KIAA1468 | 0.894 | 0.359 |  |
| ZFP41 | 0.894 | 2.254 |  |
| MTERFD2 | 0.894 | 2.254 |  |
| DERA | 0.894 | 2.254 |  |
| H3F3B | 0.894 | 2.254 |  |
| RNF13 | 0.894 | 4.899 |  |
| RANGRF | 0.894 | 5.556 |  |
| KIAA0141 | 0.893 | 0.072 |  |
| SNX29 | 0.893 | 0.179 |  |
| STUB1 | 0.893 | 0.359 |  |
| FBXO33 | 0.893 | 0.734 |  |
| LRRC58 | 0.893 | 1.379 |  |
| SLC9B2 | 0.893 | 1.379 |  |
| PSAP | 0.893 | 4.899 |  |
| SLC30A9 | 0.892 | 0.022 |  |
| SLC7A6OS | 0.892 | 0.179 |  |
| USP32 | 0.892 | 0.359 |  |
| VDAC3 | 0.892 | 0.359 |  |
| TMEM66 | 0.892 | 1.379 |  |
| KLHL22 | 0.892 | 3.394 |  |
| AFTPH | 0.892 | 4.899 |  |
| DDX24 | 0.891 | 0.072 |  |
| ZNF277 | 0.891 | 0.179 |  |
| DNAJC5 | 0.891 | 0.179 |  |
| KDM1B | 0.891 | 0.179 |  |
| FKBP8 | 0.891 | 0.359 |  |
| GNPTAB | 0.891 | 1.379 |  |
| GPR137B | 0.891 | 5.556 |  |
| HMG20A | 0.89 | 0.359 |  |
| FAR1 | 0.89 | 1.379 |  |
| ANAPC16 | 0.89 | 2.254 |  |
| LOC652276 | 0.89 | 2.254 |  |
| CHCHD1 | 0.89 | 2.254 |  |
| TRIM62 | 0.89 | 3.394 |  |
| SUGT1 | 0.89 | 3.394 |  |
| NDUFAF4 | 0.89 | 4.899 |  |
| CHORDC1 | 0.89 | 4.899 |  |
| CSRP2BP | 0.89 | 5.556 |  |
| SLC25A36 | 0.89 | 5.556 |  |
| NPEPPS | 0.889 | 0 |  |
| GZF1 | 0.889 | 0 |  |
| ESD | 0.889 | 0.072 |  |
| PMM1 | 0.889 | 0.734 |  |
| PPP1R9B | 0.889 | 0.734 |  |
| MTHFD2L | 0.889 | 0.734 |  |
| LYRM2 | 0.889 | 1.379 |  |
| ARMC1 | 0.889 | 1.379 |  |
| FLJ10038 | 0.889 | 2.254 |  |
| NEU3 | 0.889 | 3.394 |  |
| HERC2P2 | 0.889 | 5.556 |  |
| C9orf156 | 0.888 | 0.179 |  |
| GUF1 | 0.888 | 0.179 |  |
| KLHDC5 | 0.888 | 0.359 |  |
| METTL9 | 0.888 | 0.359 |  |
| RAD21 | 0.888 | 0.734 |  |
| ZBTB6 | 0.888 | 1.379 |  |
| ERI2 | 0.888 | 1.379 |  |
| NPAT | 0.888 | 1.379 |  |
| TBC1D17 | 0.888 | 2.254 |  |
| DDX42 | 0.888 | 3.394 |  |
| NRBP2 | 0.888 | 4.899 |  |
| TMEM192 | 0.887 | 0.005 |  |
| DAB2IP | 0.887 | 0.072 |  |
| UFL1 | 0.887 | 0.179 |  |
| CCDC171 | 0.887 | 3.394 |  |
| FAM173A | 0.887 | 3.394 |  |
| RPL15 | 0.887 | 3.394 |  |
| HEXDC | 0.887 | 4.899 |  |
| ACTR1A | 0.886 | 0.022 |  |
| TMEM242 | 0.886 | 0.072 |  |
| APOOL | 0.886 | 0.179 |  |
| KIF5B | 0.886 | 0.734 |  |
| MLL3 | 0.886 | 1.379 |  |
| HSP90AA1 | 0.886 | 1.379 |  |
| MTPAP | 0.886 | 1.379 |  |
| ZSWIM8 | 0.886 | 2.254 |  |
| MCEE | 0.885 | 0.072 |  |
| TSR2 | 0.885 | 0.179 |  |
| TMEM170A | 0.885 | 0.359 |  |
| PDE6D | 0.885 | 0.359 |  |
| PPAPDC2 | 0.885 | 0.734 |  |
| MIF4GD | 0.885 | 1.379 |  |
| C11orf68 | 0.885 | 3.394 |  |
| ECSIT | 0.885 | 4.899 |  |
| PCDH17 | 0.885 | 4.899 |  |
| PPP2R5C | 0.884 | 0.022 |  |
| MYO18A | 0.884 | 0.072 |  |
| ZXDB | 0.884 | 0.179 |  |
| KIAA0195 | 0.884 | 0.179 |  |
| COQ3 | 0.884 | 1.379 |  |
| GPR125 | 0.884 | 2.254 |  |
| CYP2U1 | 0.884 | 2.254 |  |
| KLC4 | 0.884 | 3.394 |  |
| DDX17 | 0.884 | 3.394 |  |
| TXNIP | 0.884 | 4.899 |  |
| C9orf91 | 0.884 | 5.556 |  |
| NICN1 | 0.883 | 0.072 |  |
| ELP4 | 0.883 | 0.359 |  |
| CYB5D2 | 0.883 | 1.379 |  |
| UBTF | 0.883 | 3.394 |  |
| SGSM2 | 0.883 | 3.394 |  |
| FAM208B | 0.883 | 4.899 |  |
| WHSC1 | 0.883 | 5.556 |  |
| VPS52 | 0.882 | 0 |  |
| ZCRB1 | 0.882 | 0.179 |  |
| SNAPC3 | 0.882 | 0.734 |  |
| DOCK4 | 0.882 | 0.734 |  |
| C14orf1 | 0.882 | 0.734 |  |
| SNX30 | 0.882 | 2.254 |  |
| MYLIP | 0.882 | 2.254 |  |
| COG2 | 0.882 | 3.394 |  |
| NUDT8 | 0.882 | 5.556 |  |
| TRAPPC12 | 0.881 | 0.005 |  |
| PPP1R26 | 0.881 | 1.379 |  |
| GBF1 | 0.881 | 1.379 |  |
| ACYP2 | 0.881 | 2.254 |  |
| DIP2B | 0.881 | 3.394 |  |
| ZNF493 | 0.881 | 4.899 |  |
| RPS6 | 0.881 | 5.556 |  |
| CACUL1 | 0.88 | 0.022 |  |
| OSBPL2 | 0.88 | 1.379 |  |
| EIF4B | 0.88 | 2.254 |  |
| MDP1 | 0.88 | 4.899 |  |
| COMTD1 | 0.88 | 4.899 |  |
| IDH3A | 0.879 | 0.072 |  |
| CDC37L1 | 0.879 | 0.179 |  |
| CHCHD6 | 0.879 | 1.379 |  |
| LOC92249 | 0.879 | 2.254 |  |
| ATP6V1B2 | 0.879 | 2.254 |  |
| MED12L | 0.879 | 3.394 |  |
| ISCU | 0.879 | 4.899 |  |
| HUWE1 | 0.878 | 0.072 |  |
| SLC25A16 | 0.878 | 0.072 |  |
| ZHX3 | 0.878 | 0.359 |  |
| HIBCH | 0.878 | 0.359 |  |
| NDUFB9 | 0.878 | 0.359 |  |
| HSF2 | 0.878 | 2.254 |  |
| TMEM178B | 0.878 | 3.394 |  |
| STIM1 | 0.878 | 3.394 |  |
| LURAP1 | 0.878 | 5.556 |  |
| GPHN | 0.878 | 5.556 |  |
| SLU7 | 0.877 | 0 |  |
| SH3BGRL | 0.877 | 0.072 |  |
| TAB1 | 0.877 | 0.072 |  |
| SFT2D1 | 0.877 | 0.179 |  |
| C15orf57 | 0.877 | 1.379 |  |
| ENTPD3-AS1 | 0.877 | 2.254 |  |
| TRAF3IP2-AS1 | 0.877 | 3.394 |  |
| VANGL2 | 0.877 | 4.899 |  |
| DHRS4 | 0.877 | 5.556 |  |
| SYNJ2BP | 0.876 | 0 |  |
| HECTD1 | 0.876 | 0 |  |
| SDHA | 0.876 | 0.022 |  |
| PIGX | 0.876 | 0.734 |  |
| BCL2L13 | 0.876 | 1.379 |  |
| TEX2 | 0.876 | 1.379 |  |
| ENDOG | 0.876 | 2.254 |  |
| HAUS6 | 0.876 | 4.899 |  |
| ZNF720 | 0.875 | 0 |  |
| ARL8B | 0.875 | 0 |  |
| RBL2 | 0.875 | 0 |  |
| GTF2H5 | 0.875 | 0.022 |  |
| VWA8 | 0.875 | 0.022 |  |
| EIF4EBP2 | 0.875 | 0.179 |  |
| PDXK | 0.875 | 0.359 |  |
| USPL1 | 0.875 | 0.359 |  |
| CALM2 | 0.875 | 0.734 |  |
| NAPEPLD | 0.875 | 2.254 |  |
| SLC22A5 | 0.875 | 2.254 |  |
| LOC100630918 | 0.875 | 3.394 |  |
| TBC1D9 | 0.875 | 3.394 |  |
| MBIP | 0.875 | 4.899 |  |
| GPR173 | 0.875 | 4.899 |  |
| NAPG | 0.874 | 0.072 |  |
| ZNF846 | 0.874 | 0.072 |  |
| UTRN | 0.874 | 3.394 |  |
| LMBRD2 | 0.874 | 3.394 |  |
| C17orf76-AS1 | 0.874 | 5.556 |  |
| RAB14 | 0.873 | 0 |  |
| PLA2G12A | 0.873 | 0 |  |
| NTPCR | 0.873 | 0.022 |  |
| KDM3B | 0.873 | 0.072 |  |
| TAOK3 | 0.873 | 0.359 |  |
| PRPS1 | 0.873 | 0.734 |  |
| CEP97 | 0.873 | 2.254 |  |
| TSNARE1 | 0.873 | 3.394 |  |
| MRPS31P5 | 0.873 | 4.899 |  |
| RNF157 | 0.873 | 4.899 |  |
| EIF4E | 0.873 | 4.899 |  |
| RIC3 | 0.873 | 4.899 |  |
| SELT | 0.872 | 0 |  |
| FBXL3 | 0.872 | 0.005 |  |
| PET112 | 0.872 | 0.022 |  |
| NOP14-AS1 | 0.872 | 0.072 |  |
| TMEM55A | 0.872 | 0.179 |  |
| ZNF280B | 0.872 | 0.359 |  |
| FBXO7 | 0.872 | 0.734 |  |
| PCGF6 | 0.872 | 3.394 |  |
| AIFM1 | 0.872 | 5.556 |  |
| HOOK3 | 0.871 | 0.005 |  |
| RMND5A | 0.871 | 0.072 |  |
| NGFRAP1 | 0.871 | 0.072 |  |
| RASA3 | 0.871 | 0.734 |  |
| AUH | 0.871 | 0.734 |  |
| TTBK1 | 0.871 | 1.379 |  |
| PAN3-AS1 | 0.871 | 2.254 |  |
| INSIG1 | 0.871 | 5.556 |  |
| SHF | 0.871 | 5.556 |  |
| C10orf76 | 0.87 | 0 |  |
| VTI1A | 0.87 | 0.005 |  |
| HIRA | 0.87 | 0.022 |  |
| EFHA1 | 0.87 | 0.072 |  |
| MAP9 | 0.87 | 0.734 |  |
| DNPH1 | 0.87 | 0.734 |  |
| ABHD4 | 0.87 | 0.734 |  |
| ILF3-AS1 | 0.87 | 1.379 |  |
| FBXW11 | 0.87 | 1.379 |  |
| LRTOMT | 0.87 | 3.394 |  |
| TPRN | 0.87 | 4.899 |  |
| LEMD3 | 0.87 | 5.556 |  |
| DEXI | 0.869 | 0 |  |
| C2orf69 | 0.869 | 0.022 |  |
| WDR48 | 0.869 | 0.022 |  |
| ZNF252P | 0.869 | 0.072 |  |
| N4BP2L2 | 0.869 | 0.072 |  |
| ZC3H7B | 0.869 | 0.072 |  |
| THEM6 | 0.869 | 0.072 |  |
| PHF10 | 0.869 | 0.734 |  |
| CAPN1 | 0.869 | 2.254 |  |
| TCEAL7 | 0.869 | 2.254 |  |
| SF1 | 0.869 | 3.394 |  |
| EAF2 | 0.869 | 4.899 |  |
| MVB12B | 0.869 | 4.899 |  |
| SLC2A6 | 0.869 | 4.899 |  |
| PPAP2B | 0.869 | 5.556 |  |
| LOC100131089 | 0.869 | 5.556 |  |
| LRPPRC | 0.868 | 0 |  |
| SMG1 | 0.868 | 0.005 |  |
| ZNF517 | 0.868 | 0.072 |  |
| IVD | 0.868 | 0.359 |  |
| DCAF11 | 0.868 | 0.359 |  |
| KTN1-AS1 | 0.868 | 2.254 |  |
| KHDRBS3 | 0.868 | 3.394 |  |
| ZNF519 | 0.868 | 4.899 |  |
| RBM17 | 0.868 | 4.899 |  |
| SENP8 | 0.868 | 5.556 |  |
| MID1IP1 | 0.868 | 5.556 |  |
| MRPS16 | 0.867 | 0 |  |
